# Downregulated Smad3 signaling impairs the maturation of MO-MDSC in colorectal cancer

**DOI:** 10.1038/s41419-025-08228-1

**Published:** 2025-12-08

**Authors:** Min Li, Xuan Zha, Zewei Gao, Yue Zhao, Jie Ma, Jie Tian, Zhenwei Mao, Shengjun Wang

**Affiliations:** 1 Department of Laboratory Medicine, Jiangsu Province Engineering Research Center for Precise Diagnosis and Treatment of Inflammatory Diseases, Affiliated Hospital, Jiangsu University, Zhenjiang, China; 2 Department of Immunology, Jiangsu University School of Medicine, Zhenjiang, China; 3 Department of Laboratory Medicine, Affiliated People’s Hospital, Jiangsu University, Zhenjiang, China

**Keywords:** Oncogenesis, Colon cancer

## Abstract

Myeloid-derived suppressor cells (MDSC) are a heterogeneous group of immunosuppressive cells that expand in tumors due to impaired differentiation into macrophages (MΦ), dendritic cells (DC), or granulocytes. Despite extensive research, the mechanisms governing their differentiation remain incompletely understood. Transforming growth factor β1 (Tgfβ1), abundant in the tumor microenvironment (TME), regulates cellular differentiation via the classical Smad2/3 pathway, yet paradoxically fails to drive MDSC maturation. In this study, we demonstrate that Smad3 is significantly downregulated in MDSC from colorectal cancer (CRC) patients and tumor-bearing mouse models, impairing monocytic lineage maturation. Myeloid-specific Smad3 overexpression promotes the differentiation of MO-MDSC into mature MΦ and DC, enhances MHC-II expression, and reduces immunosuppressive molecules, thereby attenuating tumor progression. Mechanistically, Tgfβ1 induces MO-MDSC expansion via the non-classical PI3K/AKT pathway, which is counteracted by Smad3 overexpression. Furthermore, Mettl3-mediated m^6^A modification destabilizes Smad3 mRNA at the 3’UTR, linking RNA epigenetics to MDSC dysfunction. Clinically, plasma Tgfβ1 levels correlate with the MO-MDSC/PMN-MDSC ratio across cancer types, highlighting its biomarker potential. Our findings unveil Smad3 as a critical regulator of MDSC fate and propose novel therapeutic targets for tumor immunotherapy.

## Introduction

Myeloid-derived suppressor cells (MDSC) are a heterogeneous population of immature myeloid cells comprising of myeloid progenitor cells and precursors whose normal differentiation into macrophages (MΦ), dendritic cells (DC), or granulocytes is impaired. These cells are abundantly present in the tumor microenvironment (TME) and represent one of the major obstacles limiting the efficacy of cancer immunotherapy. MDSC can be classified into two subsets based on morphological and surface marker criteria: monocytic MDSC (MO-MDSC; CD11b⁺Ly6C⁺Ly6G^−^ in mice, CD33⁺CD14⁺CD15^−^HLA-DR^−^ in humans) and polymorphonuclear MDSC (PMN-MDSC; CD11b⁺Ly6G⁺Ly6C^lo/^^−^ in mice, CD33⁺CD14^−^CD15⁺HLA-DR^−^ in humans) [1–3]. MDSC exhibits rapid responsiveness to tumor initiation, characterized by aberrant proliferation during early tumorigenesis [4–6]. Notably, MO-MDSC demonstrates superior immunosuppressive activity compared to PMN-MDSC, including stronger suppression of T cell and NK cell function, promotion of Treg differentiation, and facilitation of tumor growth and invasion across various cancer types [7, 8]. Thus, controlling MDSC expansion and/or promoting their differentiation represents a critical strategy to enhance antitumor therapy [9], underscoring the urgency to elucidate the mechanisms underlying this differentiation arrest.

Transforming growth factor β1 (Tgfβ1) plays dual roles in tumor progression [10]. During early tumorigenesis, Tgfβ1 exerts tumor-suppressive effects by inducing apoptosis, differentiation, and cell-cycle arrest through modulation of key genes such as p15, p21, c-myc, inhibitor of DNA binding (ID1/2/3), and DAP kinase [11, 12]. In advanced stages, however, Tgfβ1 acquires protumorgenic properties by driving epithelial-mesenchymal transition (EMT) and remodeling the TME into an immunosuppressive niche [13–15]. This functional dichotomy may stem from alterations in TgfβRI/II receptor expression [16] and activation of divergent downstream pathways. The canonical Tgfβ1 signaling pathway, mediated by phosphorylated Smad2/3 (p-Smad2/3), is essential for Tgfβ1-driven processes, including Treg differentiation [17, 18], osteoblastogenesis [19], myogenic differentiation [20], T cell immune response [21], and apoptosis [22–25]. Additionally, Tgfβ1 activates non-canonical pathways such as PI3K/AKT and MAPK, which are increasingly recognized for their roles in tumor progression.

Several cytokines such as GM-CSF, IL-6, IFN-γ, and IL-1β have been identified as inducers of tumor MDSC [26]. Studies showed that Tgfβ1 promoted the expansion of MO-MDSC and enhanced their immunosuppressive functions [13]. It is pertinent for us to analyze the function of Tgfβ1 in tumor MDSC, a growth factor abundant in tumors and possessing the ability to promote cell differentiation, which did not help the differentiation of MDSC into mature cells.

In this study, we systematically elucidated the expression and activation characteristics, as well as the functional roles, of the classical Smad2/3 signaling pathway and the non-classical PI3K/AKT signaling pathway in the context of tumor MO-MDSC differentiation blockade and expansion, suggesting that the influence of Tgfβ1 on the maturation and expansion of MO-MDSC is not uniform but variable and subject to regulation. Furthermore, we delineated the critical role of Mettl3-mediated m^6^A RNA epigenetic modifications in Tgfβ1-modulated cell fate determination through the regulation of Smad3 expression.

## Results

### The role of Smad3 in promoting monocytic differentiation and its downregulation in tumor MDSC

**Fig. 1:**
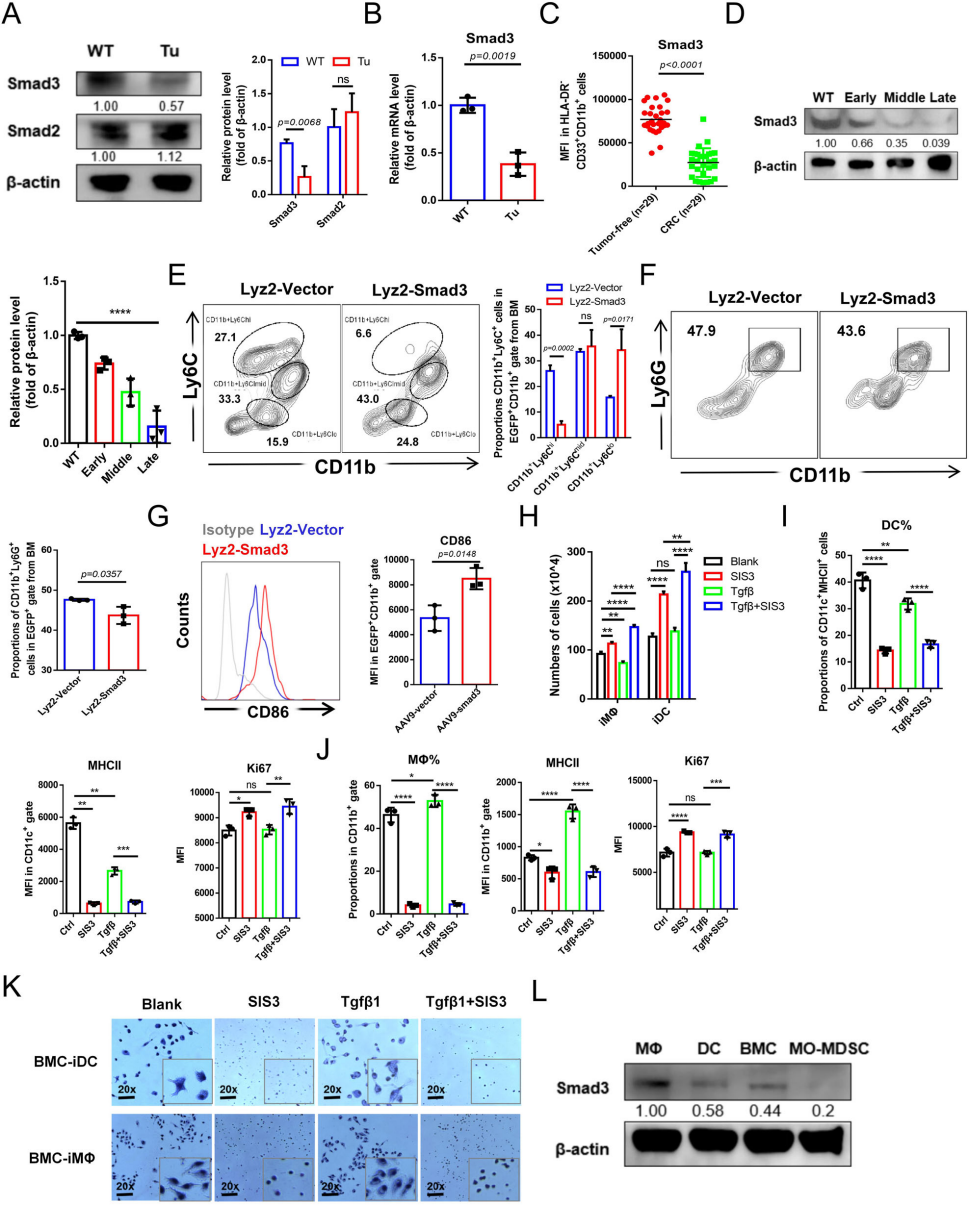
The role of Smad3 in promoting monocytic differentiation and its downregulation in tumor MDSC. **A** Western blot analysis of the Smad2/3 expressions in wide-type-derived CD11b^+^Gr-1^+^ cells (WT) and CT26-derived spleen CD11b^+^Gr-1^+^ MDSC (Tu). **B** Quantification of relative Smad3 transcription levels in wide-type-derived CD11b^+^Gr-1^+^ cells (WT) and CT26-derived spleen CD11b^+^Gr-1^+^ MDSC (Tu). **C** FCM analysis of the Smad3 expressions in HLA-DR^−^CD33^+^CD11b^+^MDSC derived from peripheral blood of CRC patients (*n* = 30) and healthy donors (*n* = 29). **D** Western blot analysis of the Smad3 expressions in wide-type-derived CD11b^+^Gr-1^+^ cells (WT) and MDSC with tumor progression of CT26 mouse models respectively at early (8 d), middle (18 d), and late stage (28 d). **E** FCM analysis of the proportions of CD11b^+^Ly6C^hi^, CD11b^+^Ly6C^mid^, and CD11b^+^Ly6C^lo^ cells in the EGFP^+^CD11b^+^ cells of bone marrow from AAV9-Smad3/vector group of mice. **F** FCM analysis of the proportions of CD11b^+^Ly6G^hi^ cells in the EGFP^+^CD11b^+^ cells of bone marrow from AAV9-Smad3/vector group of mice. **G** FCM analysis of the expressions of CD86 in the EGFP^+^CD11b^+^ cells of bone marrow from AAV9-Smad3/vector group of mice. **H** Cell numbers in control group, 2 μM SIS3 treatment group, 20 ng/mL Tgfβ1 treatment group and Tgfβ1 + SIS3 treatment group after MΦ induction by 20 ng/mL M-CSF for 7 days and DC induction by 20 ng/mL GM-CSF and 10 ng/mL IL-4 for 9 days. **I**–**K** Analyzing DC and MΦ proportions, MHCII and Ki67 expressions, and cellular morphology of each treatment group respectively in DC and MΦ induction system. **L** Western blot analysis of the Smad3 expressions in initial bone marrow cells (BMC) and BMC-induced MΦ, DC, and MO-MDSC. Except C (*n* = 29), error bars of statistical graphs represent mean ± SD, *n* = 3. For (**A**–**C**, **E**–**G**), *p*-values were determined using two sides unpaired t-test; For (**D**, **H**–**J**), *p*-values were determined using one-way ANOVA with Tukey test. *p < 0.05; **p < 0.01; ***p < 0.001; ****p < 0.0001; ns no significant.

We first assessed the abundance of Smad2/3 signaling in tumor MDSC derived from CT26 tumor-bearing mice. No significant difference in Smad2 expression was observed. However, Smad3 expression was significantly downregulated in CD11b^+^Gr-1^+^ tumor MDSC, which exhibited immunosuppressive functions and elevated levels of immunosuppressive molecules such as arginase-1 (Arg-1), inducible nitric oxide synthase (iNOS), and reactive oxygen species (ROS), compared to wild-type cells (Fig. 1A and Supplementary Fig. 1A–C). Smad3 transcript levels were also reduced (Fig. 1B). Line with these findings, MDSC from colorectal cancer (CRC) patients displayed lower Smad3 expression than those from healthy donors (Fig. 1C and Supplementary Fig. 1D, Table S1). It was hypothesized whether Tgfβ1 failed to promote MDSC maturation as a result of the limited expression of Smad3. Indeed, Smad3 levels in MDSC decreased at early-stage tumors (the diameter of tumor tissue was between 5 and 8 mm on the 8th day of CT26 model construction) and further as tumors progressed (Fig. 1D). To further decipher the correlation of Smad3 abundance with MDSC differentiation, Smad3 expression in bone marrow cells (BMC)-induced MDSC was analyzed. Consistently, both protein and transcription levels of Smad3 were obviously reduced in bone marrow-induced MDSC (BM-MDSC) compared to primary BMC (Supplementary Fig. 1E, F).

Smad3 has been reported important roles in cell differentiation of regulatory T cells, osteoblasts, and myoblasts [17–20], but comprehensive studies on its involvement in myeloid differentiation are still limited. To clarify the role of Smad3 in normal myelopoiesis, we generated mice with myeloid-specific Smad3 overexpression (Lyz2-Smad3) by Lyz2>Kozak-Smad3/EGFP-AAV9 construction. After 4 weeks, strong EGFP fluorescence in both limbs was observed, with significantly increased Smad3 levels in EGFP^+^CD11b^+^ BMC of Lyz2-Smad3 group of mice (Supplementary Fig. 1G–I). Noticeably, these mice showed significant decreases in CD11b^+^Ly6C^hi^ cells and CD11b^+^Ly6G^+^ cells, accompanied by a marked increase in CD11b^+^Ly6C^lo^ cells within BM (Fig. 1E, F). As previously established, CD11b^+^Ly6C^lo^ cells present more mature monocytic characteristics than Ly6C^hi^ cells [27–29]. Indeed, EGFP^+^CD11b^+^ cells within BM of Lyz2-Smad3 mice exhibited higher expression of CD86, a maturation marker of monocytic lineage (Fig. 1G). Moreover, in the spleen, higher proportions of both CD11b^+^F4/80^+^ MΦ and CD11c^+^MHCII^+^ DC were observed in the Lyz2-Smad3 group of mice under EGFP^+^ gate, along with fewer CD11b^+^Ly6G^+^ granulocytes (Supplementary Fig. 1J). No significant shift in proportions of MHCII^+^CD206^-^ M1 and MHCII^−^CD206^+^M2 was detected, whereas almost all of these cells expressed the MHCII^+^CD206^-^ M1 phenotype (Supplementary Fig. 1K). All these results suggest a potential role of Smad3 in monocytic development.

We then confirmed the positive role of Smad3 in the monocytic maturation into MΦ and DC in vitro. Minimal autocrine Tgfβ1 was detected across all myeloid differentiation processes (Supplementary Fig. 1L). As a canonical downstream of Tgfβ1, a specific inhibitor of Smad3 activation, SIS3, was employed to further investigate the role of Tgfβ1/Smad3 pathway in monocytic lineage development. Strikingly, SIS3 treatment triggered a marked proliferation and impaired maturation, as shown by higher cell counts, elevated Ki67 expression, and fewer MΦ and DC proportions along with lower MHCII expressions (Fig. 1H–J). Correspondingly, SIS3-treated cells exhibited a high cytoplasmic ratio and the absence of synapses, characteristics of immature morphology (Fig. 1K). In addition, Tgfβ1 suggested a facilitation of MΦ maturation with upregulated MHCII. However, those were largely reversed upon SIS3 treatment (Fig. 1H–K), indicating a Smad3-dependent manner in Tgfβ1-mediated monocytic maturation. Therefore, we hypothesized that the impaired MO-MDSC maturation is likely correlated with the absence of Smad3. In fact, Smad3 levels were higher during MΦ and DC differentiation, but significantly lower in MO-MDSC than those in bone marrow-derived MΦ, DC, and peritoneal macrophages (pMΦ) (Supplementary Fig. 1M–O and Fig. 1L).

### Smad3 overexpression promotes MO-MDSC maturation and attenuates tumor growth

**Fig. 2:**
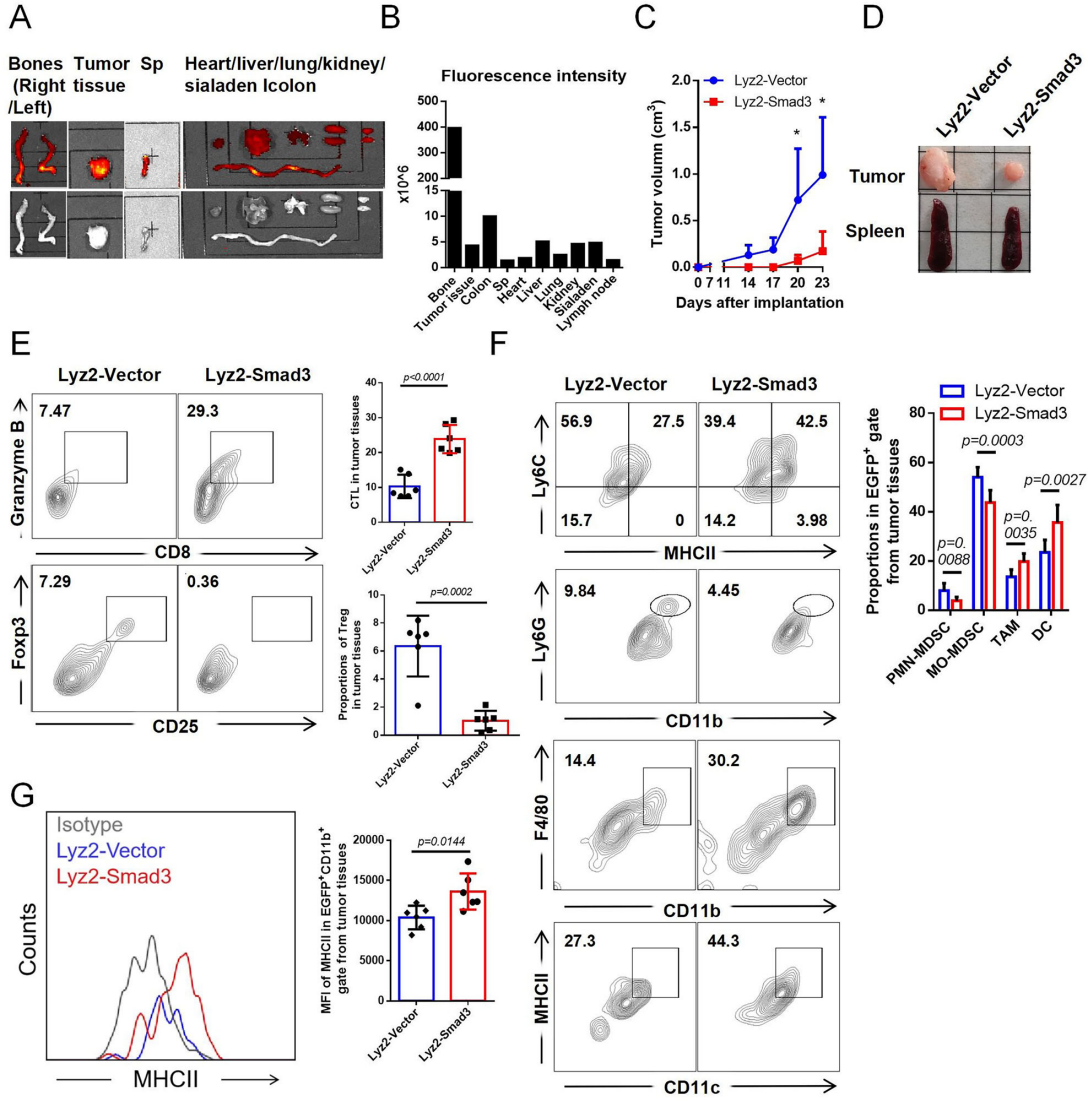
Myeloid-Smad3 overexpression attenuates tumor growth and drives monocytic maturation. **A**, **B** Animal imaging analysis of the fluorescence intensity and distribution of EGFP in the organs of mice with injection of Lyz2>Kozak-Smad3-AAV9 and construction of CT26 mouse models for 4 weeks. **C** Analysis of the tumor growth curve in mice with injection of Lyz2>Kozak-Smad3/EGFP-AAV9 (Lyz2-Smad3 group) or Lyz2>Kozak/EGFP-AAV9 (AAV9-Vector group) during construction of CT26 mouse models for 24 days. **D** Analysis of the tumor and spleen size in Lyz2-Smad3/Vector group mice after construction of CT26 mouse models for 24 days. **E** FCM analysis of the proportion of CTL and Treg cells infiltrated in tumor tissues in Lyz2-Smad3/Vector group mice. **F** FCM analysis of the proportion of CD11b^+^Ly6G^hi^ PMN-MDSC, CD11b^+^Ly6C^hi^ MO^−^MDSC, CD11b^+^F4/80^hi^ MΦ, and CD11c^+^MHCII^+^ DC infiltrated in tumor tissues in Lyz2-Smad3/Vector group mice under EGFP^+^ gate. **G** FCM analysis of the MHCII expression in EGFP^+^ CD11b^+^ cells in tumor tissues in Lyz2-Smad3/Vector group mice. For (**C**, **E**–**G**), *n* = 6. *p*-values were determined using two sides unpaired t-test. *p < 0.05.

We next examined whether enhancing Smad3 expression could rescue MO-MDSC maturation in tumor-bearing Lyz2-Smad3 mice. Smad3^+^ myeloid cells were enriched in BM and also detected in tumors and other organs such as colons, livers, kidneys, and salivary glands (Fig. 2A, B). Remarkably, Lyz2-Smad3 mice showed slow tumor growth and reduced splenomegaly (Fig. 2C, D). Tumors and spleens from these mice contained more cytotoxic CD8^+^ T cells and fewer Tregs (Fig. 2E and Supplementary Fig. 2A). Importantly, CD11b^+^MHII^-^Ly6C^hi^ MO-MDSC and CD11b^+^Ly6G^hi^ PMN-MDSC in tumor tissues were substantially reduced in Lyz2-Smad3 mice within EGFP^+^ gates, as well as in spleens and peripheral blood (Fig. 2F and Supplementary Fig. 2B, C). In contrast, CD11b^+^F4/80^+^ MΦ and CD11c^+^MHCII^+^ DC were significantly increased (Fig. 2F and Supplementary Fig. 2D). Consistent with that in tumor-free mice, M1/M2 ratios in tumor tissues and spleens remained unchanged within EGFP^+^CD11b^+^F4/80^+^ gates (Supplementary Fig. 2E, F). Tracing origins, BM analysis revealed fewer Ly6C^hi^ MO-MDSC but more Ly6C^lo^ mature cells [8] in Lyz2-Smad3 mice within EGFP^+^CD11b^+^ gates (Supplementary Fig. 2G). MHCII expression was elevated in tumor-infiltrating and BM-derived EGFP^+^CD11b^+^ myeloid cells (Fig. 2G and Supplementary Fig. 2H). Therefore, these findings suggest a crucial role of Smad3 in monocytic maturation and limiting frequencies of MO-MDSC in tumor development.

**Fig. 3:**
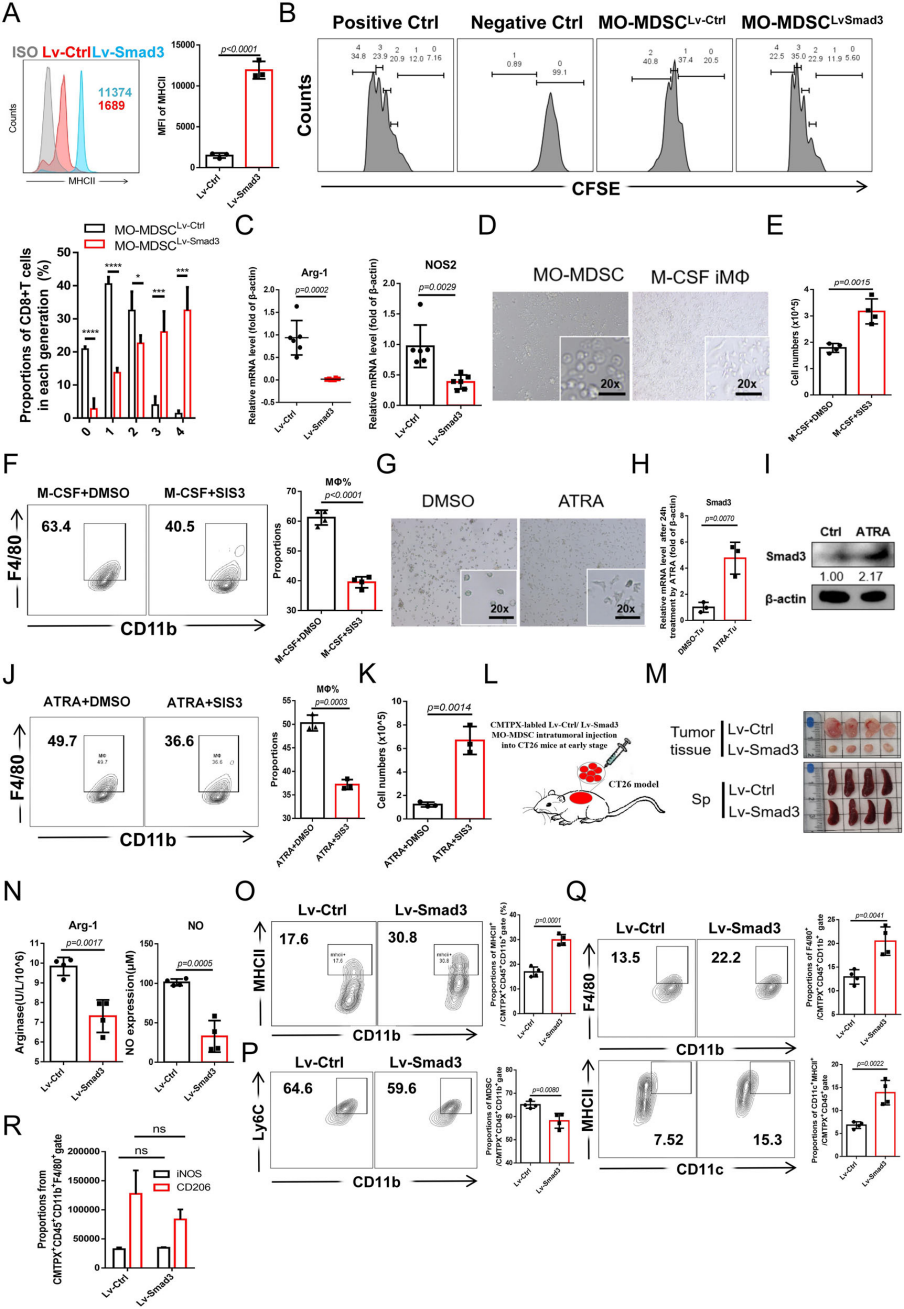
Smad3 overexpression promotes MO-MDSC maturation and attenuates tumor growth. **A** FCM analysis of the MHCII expressions after lentivirus transfection of Smad3/Ctrl (Lv-Smad3/Lv-Ctrl) in tumor MO-MDSC for 5 days within TCCM condition. **B** Detection of suppressive function of MO-MDSC after Smad3 overexpression to CD8^+^T cell proliferation isolated from naïve mice by CFSE labeling assay. **C** qRT-PCR analysis of the Arg-1 and NOS2 expressions in tumor MO-MDSC after lentivirus-transfection of Smad3/Ctrl (Lv-Smad3/Lv-Ctrl) for 24 h within TCCM condition. **D** Cell morphology of tumor MO-MDSC treated with or without 2500 U/mL M-CSF for 5 days. **E**, **F** FCM analysis of the effects of 2 μM DMSO/SIS3 to the matured MΦ from tumor MO-MDSC within 2500U/mL M-CSF condition for 5 days and cell numbers. **G** Cell morphology of tumor MO-MDSC treated with or without 2 μM ATRA, 20% TCCM, and 10 ng/mL GM-CSF for 5 days. **H**, **I** qRT-PCR and Western blot analysis of the Smad3 expressions in tumor MO-MDSC treated with 2 μM DMSO/ATRA for 24 h and 48 h respectively. **J**, **K** FCM analysis of the effects of DMSO/SIS3 to the matured MΦ from tumor MO-MDSC within 2 μM ATRA, 20% TCCM and 10 ng/mL GM-CSF for 5 days and cell numbers. **L** Diagram of intra-tumoral injection of CMTPX probe labeled 5 × 10^6^ Lv-Smad3/Lv-Ctrl tumor MO-MDSC in early CT26 mice (day 8 post-inoculation, tumor volume variation <1 mm^3^). **M** Sizes of tumors and spleens in Lv-Smad3/Lv-Ctrl group of mice post five days of adoptive transfer. **N** Detections of Arg-1 activity and NO concentrations of CMTPX^+^ cells isolated by flow cytometry sorting from tumor tissues of Lv-Smad3/Lv-Ctrl group of mice post five days of adoptive transfer. **O** FCM analysis of the proportions of MHCII^+^ cells in CMTPX^+^CD45^+^CD11b^+^ gate in tumor tissues after Lv-Smad3/Lv^-^Ctrl tumor MO-MDSC injected for 5 days. **P**, **Q** FCM analysis of the proportions of MDSC and F4/80^+^ cells in CMTPX^+^CD45^+^CD11b^+^ gate^,^ and CD11c^+^MHCII^+^ DC in CMTPX^+^CD45^+^ gate in tumor tissues after Lv-Smad3/Lv^−^Ctrl tumor MO-MDSC injected for 5 days. **R** FCM analysis of the expressions of CD206 and iNOS within CMTPX^+^CD45^+^CD11b^+^F4/80^+^ cells in tumor tissues after Lv-Smad3/Lv^−^Ctrl tumor MO-MDSC injected for 5 days. For (**A**, **H**, **J**, **K**), *n* = 3; For (**B**, **E**, **F**, **N**, **O**, **Q**, **R**), *n* = 4; For (**C**), *n* = 6. *p*-values were determined using two sides unpaired t-test. *p < 0.05; ***p < 0.001; ****p < 0.0001; ns no significant.

To directly test the effect of Smad3 on MO-MDSC maturation, we overexpressed Smad3 in tumor-derived highly immunosuppressive MO-MDSC via lentiviral transduction. Smad3-overexpressing MO-MDSC showed higher MHCII expression and gave rise to more MΦ within CT26 cell-conditioned medium (TCCM) culture condition for 5 days (Supplementary Fig. 3A, Fig. 3A, and Supplementary Fig. 3B). It also exhibited almost disappeared immunosuppressive function, with decreased Arg-1 and NOS2 expression and loss of CD8^+^T cell suppression capacity (Fig. 3B, C). In a MΦ differentiation system driven by M-CSF from tumor MO-MDSC where approximately 50% of cells transformed (Fig. 3D), Smad3 levels significantly increased during maturation (Supplementary Fig. 3C, D). Upon inactivation of Smad3 by SIS3, total cell numbers were increased (Fig. 3E) with a substantial decrease in MΦ frequency (Fig. 3F). Similar effects were confirmed in MΦ differentiation driven by M-CSF from BM-MO-MDSC under Smad3 inactivation or overexpression (Supplementary Fig. 3E–G). All-trans retinoic acid (ATRA), a known agent intended to specifically “push” MDSC differentiation [30], caused significant morphological changes in tumor MO-MDSC, transitioning them into spindle shapes after 5 days (Fig. 3G). ATRA notably increased Smad3 expression (Fig. 3H, I) and showed at least partial reliance on Smad3 (Fig. 3J, K), which was also observed in lewis tumor models (Supplementary Fig. 3H–J). Given the difficulty of driving tumor MO-MDSC into DC in vitro, we determined the role of Smad3 in BM-MO-MDSC maturation into DC. Similar to the MΦ induction results, SIS3 treatment reduced DC induction (Supplementary Fig. 3K, L), while Smad3 overexpression increased DC proportions and MHCII expression (Supplementary Fig. 3M, N). Thus, boosting Smad3 expression directly drives the maturation of tumor MO-MDSC into MΦ and DC in vitro.

We then verified the reliability by intratumoral adoptive transfer of CMTPX probe-labeled MO-MDSC^Lv-Smad3/Ctrl^ at early-stage tumors (Fig. 3L). MO-MDSC^Lv-Smad3^ transfer group suggested much smaller tumors and spleens (at 5th day post transfer) (Fig. 3M), indicating a really improved ability to combat tumors. To elucidate whether it is correlated with weakened immunosuppressive functionality and decreased frequency as a result of maturation level changes of MO-MDSC, CMTPX^+^cells from tumor tissues were further examined. Actually, CMTPX^+^ cells isolated from Lv-Smad3 MO-MDSC transfer group by flow cytometry sorting five days post-injection suggested marked decreases in Arg-1 activity and NO secretions (Fig. 3N). Moreover, CMTPX^+^CD45^+^cells from MO-MDSC^Lv-Smad3^ transfer group showed significantly increased MHC II expressions accompanied by decreased CD11b^+^Ly6C^hi^ cells and notable increased F4/80^+^ MΦ and CD11c^+^MHCII^+^ DC (Fig. 3O–Q). No change was observed in M1/M2 ratio for MO-MDSC-matured MΦ (Fig. 3R). These results confirm that Smad3 deficiency underlies defective MO-MDSC maturation.

### Smad3 overexpression counteracts Tgfβ1-induced MO-MDSC expansion

**Fig. 4:**
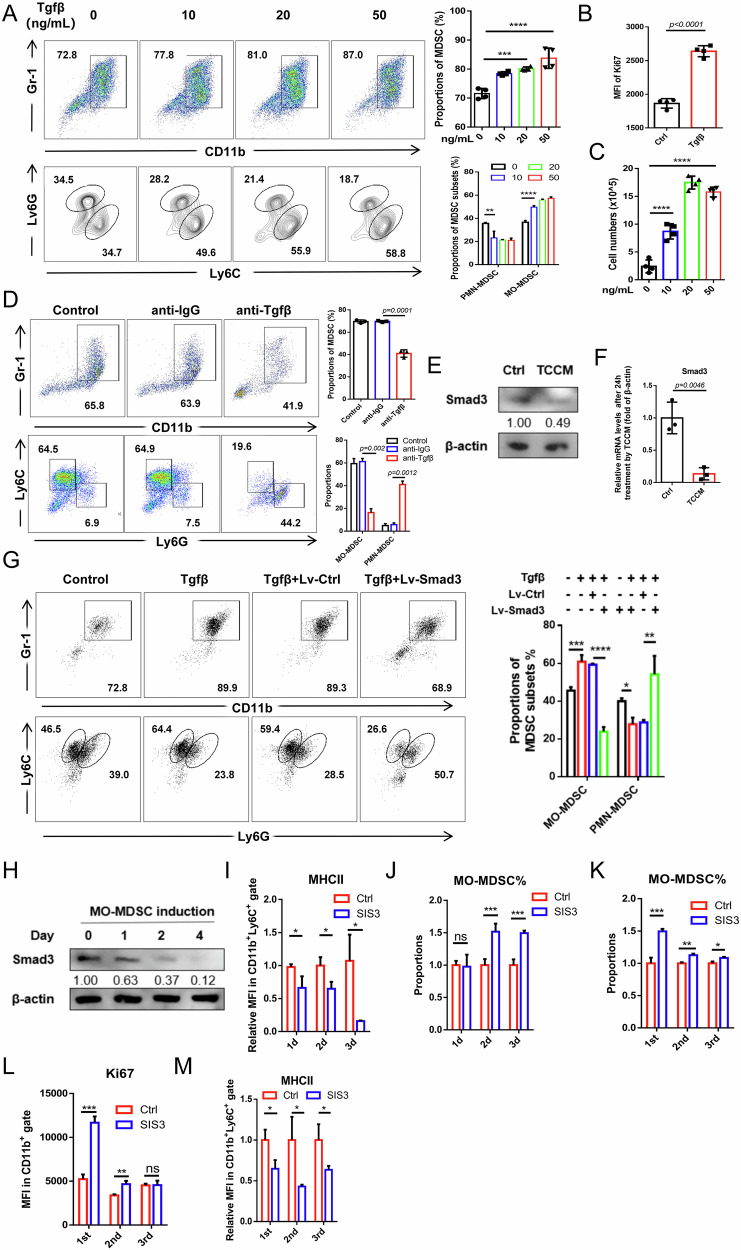
Smad3 overexpression counteracts Tgfβ1-induced MO-MDSC expansion. **A** FCM analysis of the proportions of MDSC and subgroups after 0/10/20/50 ng/mL Tgfβ1 treatment in MDSC induction from BMC by 20 ng/mL GM-CSF and 20 ng/mL IL-6 for 4 days. **B** FCM analysis of the Ki67 expressions in 0 (Ctrl) or 10 ng/mL Tgfβ1(Tgfβ) induced MDSC combined with GM-CSF and IL-6 for 4 days. **C** Cell numbers of MDSC induction system from BMC by GM-CSF and IL-6 after 0/10/20/50 ng/mL Tgfβ1 treatment for 4 days. **D** FCM analysis of the proportions of MDSC and subgroups with or without 10 μM anti-IgG/Tgfβ1 in MDSC induction from BMC by 50% TCCM and 20 ng/mL GM-CSF. **E**, **F** Western blot and qRT-PCR analysis of the Smad3 expression in BMC with or without TCCM treatment for 48 hours and 24 h respectively. **G** FCM analysis of proportions of MDSC subsets after Tgfβ1 treatment with or without lentivirus-transfection of Smad3/Ctrl (Lv-Smad3/Lv-Ctrl) in MDSC induction by GM-CSF and IL-6. **H** Western blot analysis of the expressions of Smad3 during MO-MDSC induction from BMC by 50% TCCM and 20 ng/mL GM-CSF at 0, 1, 2, and 4 days. **I**, **J** Monitoring MHCII expressions and MO-MDSC proportions by FCM during BM-MO-MDSC induction under 50% TCCM and 20 ng/mL GM-CSF after SIS3 treatment for 1, 2, and 3 days. (**K**–**M**) FCM analysis of MO-MDSC proportions, Ki67, and MHCII expressions at the 4th day in each group that treated with SIS3 at the 1st, 2nd, and 3rd day of MO-MDSC induction under 50% TCCM and 20 ng/mL GM-CSF. For (**A**–**C**), *n* = 4; For (**D**, **F**, **G**, **I**–**M**), *n* = 3. For (**A**, **C**, **D,** **G**), *p*-values were determined using one-way ANOVA with Tukey test; For (**B**, **F**, **I**–**M**), *p*-values were determined using two sides unpaired t-test. *p < 0.05; **p < 0.01; ***p < 0.001; ****p < 0.0001; ns no significant.

Previous studies implied that Tgfβ1 could promote the expansion and function of MO-MDSC [13, 31]. To elucidate the roles of Tgfβ1/Smad3 pathway in MDSC expansion, Tgfβ1 were introduced into BM-MDSC induction system. Indeed, Tgfβ1 promoted MO-MDSC expansion in a dose-dependent manner under both kinds of induction system in vitro, along with increased Ki67 expression and cell counts (Fig. 4A–C and Supplementary Fig. 4A–C). Moreover, the vital role of Tgfβ1 in TCCM-mediated MO-MDSC expansion was also indicated by anti-Tgfβ1 treatment (Fig. 4D and Supplementary Fig. 4D). Interestingly, statistical increases of both proportions and counts of PMN-MDSC were observed upon anti-Tgfβ1 treatment but not significant after Tgfβ1 append within TCCM conditions, which might involve an altered cytokine kinetics and precursor competition that the abundant pro-granulocytic or PMN-MDSC factors (e.g., G-CSF, IL-6, GM-CSF, CCLs) within TCCM acted unopposed upon Tgfβ1 neutralized [32–34]. Notably, both protein and transcription levels of Smad3 were downregulated by TCCM treatment (Fig. 4E, F).

Therefore, it was hypothesized that the facilitation of Tgfβ1 in MO-MDSC expansion is not via the Smad3 pathway. Indeed, boosting Smad3 expression suggested a reverse of Tgfβ1-induced BM-MO-MDSC expansion within GM-CSF and IL-6 condition (Fig. 4G). Moreover, Smad3 overexpression reversed BM-MO-MDSC expansion within TCCM condition with a notable increase in MHCII expression (Supplementary Fig. 4E, F). Conversely, Smad3 inhibition increased MO-MDSC expansion (Supplementary Fig. 4G–I). We next identify whether Smad3’s impact on monocytic differentiation is dependent on Tgfβ1 via Tgfβ1 neutralizing. Results showed that the effect of Smad3 on MO-MDSC expansion was Tgfβ1-dependent (Supplementary Fig. 4J), though not during DC and MΦ differentiation (Supplementary Fig. 4K, L), which might indicate other potential factors besides Tgfβ1 activating the Smad3 pathway. Taken together, these results demonstrated that Tgfβ1-induced MO-MDSC expansion is not via Smad3 pathway and can be counteracted by Smad3 overexpression in a Tgfβ1-dependent manner.

To deeply understand the role of Smad3 in MO-MDSC expansion, time-course analysis was conducted. It was shown that Smad3 protein decreased early during MO-MDSC induction (Fig. 4H). SIS3 treatment from day 1 onward impaired differentiation (MHCII expression) and increased MO-MDSC proportions in a time-dependent manner (Fig. 4I, J). Later SIS3 treatment had less effect on expansion but could still enhance maturation (Fig. 4K–M). Thus, Smad3 restrains MO-MDSC expansion and promotes maturation throughout the differentiation process.

### PI3K/AKT signaling mediates Tgfβ1-driven MO-MDSC expansion

**Fig. 5:**
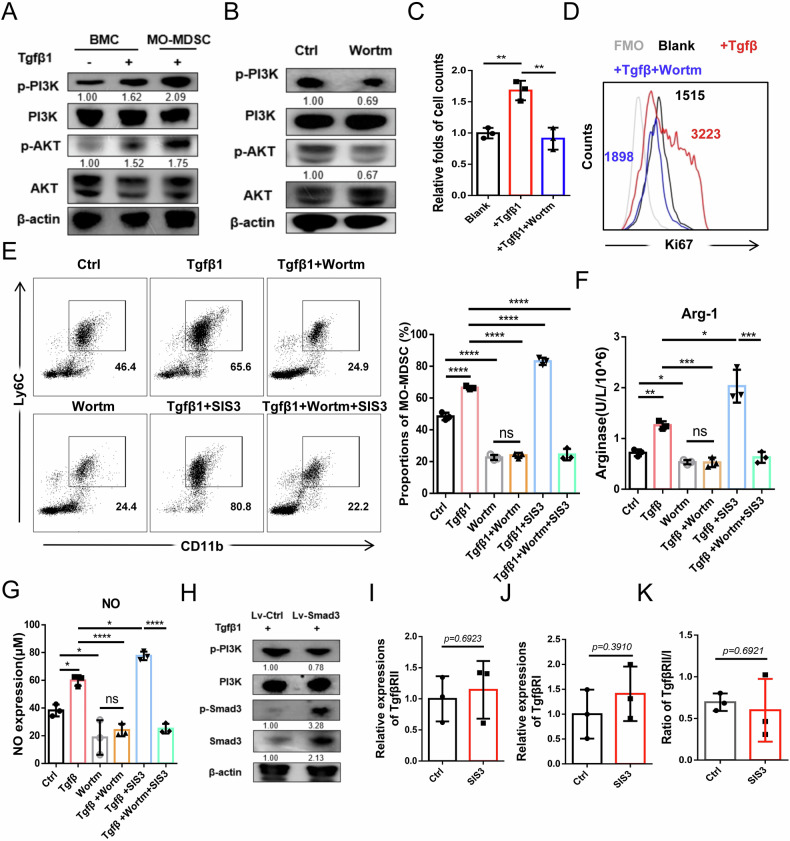
PI3K/AKT signaling mediates Tgfβ1-driven MO-MDSC expansion. (**A**) Western blot analysis of the activations of PI3K/AKT pathway in BMC and BM-induced MO-MDSC by TCCM with or without 20 ng/mL Tgfβ1 treatment for 2 hours. (**B**) Western blot analysis of the activations of PI3K/AKT pathway in BM-induced MO-MDSC with 0.2 μM DMSO/Wortm treatment for 2 hours under Tgfβ1 condition. (**C**, **D**) FCM analysis of cell numbers and Ki67 expressions in BMC treated with or without 0.2 μM DMSO/Wortm under Tgfβ1 condition for 48 hours. (**E**) FCM analysis of the proportions of induced MO-MDSC from BMC by GM-CSF and IL-6 for 4 days treated with or without 20 ng/mL Tgfβ1/ 0.2 μM Wortm/ 2 μM SIS3. (**F**, **G**) ELISA analysis of Arg-1 and NO concentrations in each group. (**H**) Western blot analysis of the PI3K activation in BM-induced MO-MDSC after Lv-Ctrl/Smad3 transfection for 48 hours under Tgfβ1 condition. (**I**–**K**) FCM analysis of the TgfβRII and TgfβRI expression and TgfβRII/I ratio in BM-MO-MDSC treated with 2 μM DMSO/SIS3 for 48 hours. Error bars of statistic graphs represent mean ± SD, n = 3. For **C** and **E**–**G**, *p*-values were determined using one-way ANOVA with Tukey test; For **I**–**K**, *p*-values were determined using two sides unpaired t-test. *p < 0.05; **p < 0.01; ****p < 0.0001; ns no significant.

We next asked how Tgfβ1 promotes MO-MDSC expansion despite impaired Smad3 signaling. PI3K/AKT, a non-canonical Tgfβ1 pathway with known pro-proliferative effects via c-myc, has been identified to be closely related to the expansion of tumor MDSC [35–37]. It was surprisingly found that PI3K/AKT signaling was robustly activated by Tgfβ1 in initial BMC and more strongly in BM-MO-MDSC (Fig. 5A). We thus hypothesized that PI3K/AKT was involved in Tgfβ1-induced MO-MDSC proliferation. As expected, inhibition of PI3K/AKT with wortmannin abolished Tgfβ1-induced proliferation (Fig. 5C, D). Induced MO-MDSC by Tgfβ1 were also surprisingly almost reversed following wortmannin treatment (Fig. 5E). Given the nearly entirely lost capacity of Tgfβ1 to induce MO-MDSC expansion upon PI3K/AKT inactivation, we tried to investigate whether MO-MDSC expansion and cell proliferation following Smad3 inhibition, as demonstrated in Fig. 4J–L, was also associated with PI3K/AKT pathway. Wortmannin was used in the BM-MO-MDSC induction system before SIS3 treatment, and it was found that SIS3 was no longer capable of affecting MO-MDSC expansion (Fig. 5E–G). Thus, it is reasonably hypothesized that the inhibition of Smad3 enhanced PI3K/AKT activation, which in turn promoted MO-MDSC proliferation. Indeed, reduced activation of PI3K/AKT was observed upon Smad3 overexpression in BM-MO-MDSC (Fig. 5H). This interestingly suggests that when the Tgfβ1/Smad3 pathway, which is responsible for differentiation, is highly activated, a concomitant reduction in the activation of Tgfβ1/PI3K/AKT pathway will be developed, which is associated with proliferation. Moreover, the proliferation-promoting effects of Smad3 were also confirmed in a Tgfβ1-dependent manner in normal DC and MΦ development (Supplementary Fig. 5A, B). Research has shown that the balance between the classical and non-classical pathways of Tgfβ1 could be regulated by the Tgfβ receptor, specifically the TgfβRII/I ratio. Nevertheless, no changes in expressions of TgfβRII (Fig. 5I), TgfβRI (Fig. 5J), or the TgfβRII/I ratio (Fig. 5K) were observed after a 48-hour SIS3 treatment. In conclusion, these findings highlight the crucial roles of PI3K/AKT signaling in the Tgfβ1-induced expansion of MO-MDSC and indicate a wonderful balance of Tgfβ1 in cell differentiation or proliferation through distinct downstream pathways.

### Mettl3/m^6^A/Ythdf2-mediated mRNA decay underlies Smad3 suppression in MO-MDSC

Consistent with the results for total MDSC, transcripts of Smad3 suggested a significant decrease in tumor MO-MDSC compared to wild-type mice-derived CD11b^+^Ly6C^+^ cells and pMΦ, which prompted us to investigate the underlying mechanisms (Supplementary Fig. 6A). Post-transcriptional epigenetic modification is a critical mechanism for regulating gene transcription through modulation of RNA transcription, stability, and translation. Therefore, we considered possible epigenetic modifications of Smad3 transcripts. According to RMBase 2.0 database (https://rna.sysu.edu.cn/rmbase/), minor modifications, such as m^5^C, m^1^A, and 2’O-Me, were predicted for Smad3 transcripts except multiple N^6^-methyladenosine (m^6^A) sites in both human and mouse samples, including two in the 3′UTR (Supplementary Fig. 6B). m^6^A has been identified as the most prevalent internal modification in mRNA with wide effects on RNA stability and translation efficiency [38]. Therefore, the effect of m^6^A modification on Smad3 mRNA expression was next investigated.

**Fig. 6:**
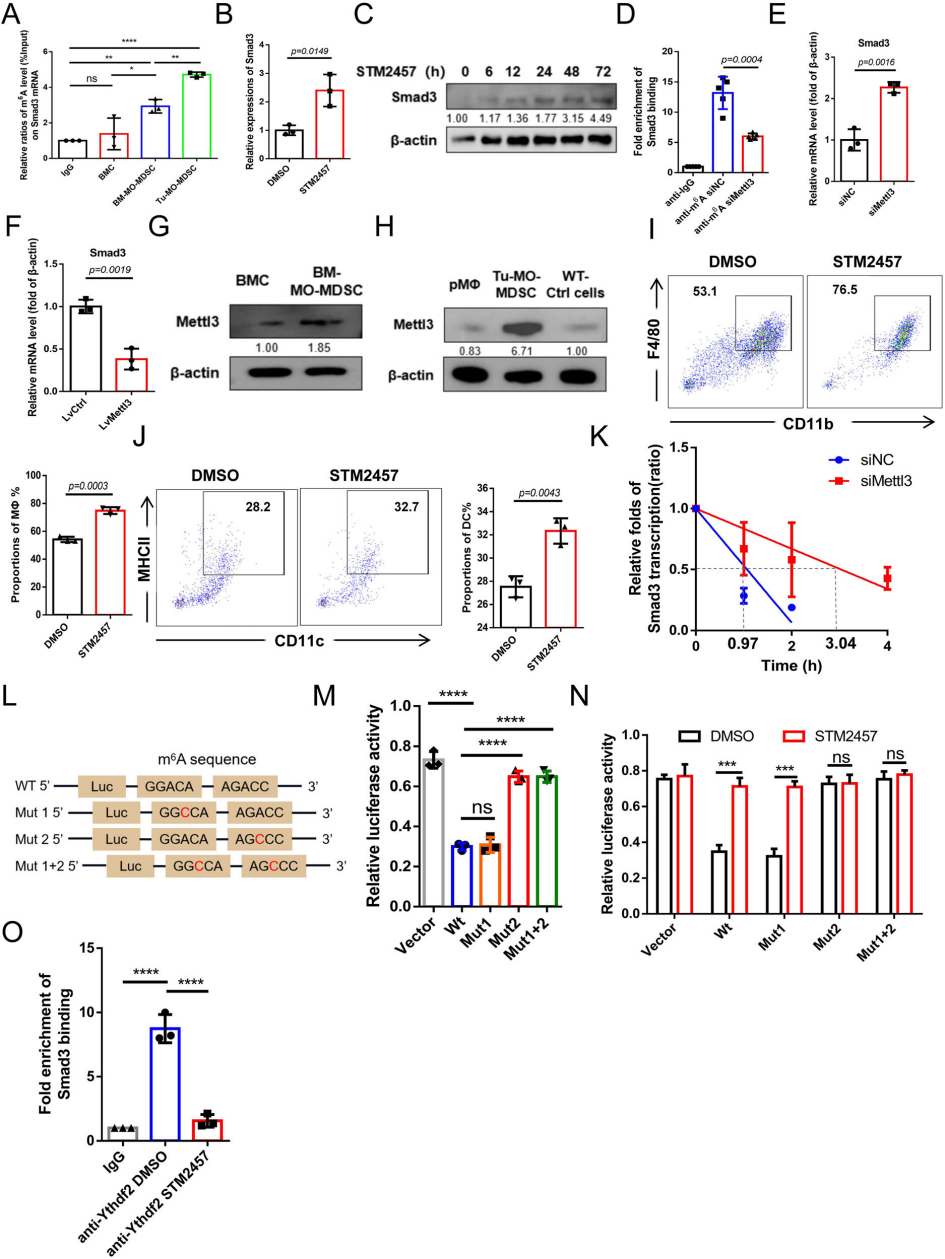
Mettl3/m^6^A-mediated mRNA decay underlies Smad3 suppression in MO-MDSC. (**A**) MeRIP-qPCR analysis of the m^6^A levels on Smad3 mRNA in BMC, BM-MO-MDSC and MO-MDSC derived from CT26 tumor models. (**B**) qRT-PCR analysis of the Smad3 expressions in tumor MO-MDSC treated with 5 μM DMSO/STM2457 for 24 hours. (**C**) Western blot analysis of the Smad3 expressions in tumor MO-MDSC with or without 5 μM STM2457 treatment for 6/12/24/48/72 hours. (**D**) MeRIP-qPCR analysis of the m^6^A levels on Smad3 mRNA in tumor MO-MDSC under siNC/siMettl3 treatment for 48 hours. (**E**) qRT-PCR analysis of the Smad3 expressions in tumor MO-MDSC treated with siNC/siMettl3 for 48 hours. (**F**) qRT-PCR analysis of the Smad3 expressions in tumor MO-MDSC treated with Lv-Ctrl/Lv-Smad3 for 48 hours. (**G**) Western blot analysis of the Mettl3 expression in initial bone marrow cells (BMC) and bone marrow induced MO-MDSC by TCCM (BM-MO-MDSC). (**H**) Western blot analysis of the Mettl3 expressions in wide-type mice-derived pMΦ, tumor-derived MO-MDSC and wide-type mice-derived CD11b^+^Ly6C^+^ cells. (**I**) FCM analysis of the proportions of MΦ after 5 μM DMSO/STM2457 treatment for 5 days of tumor MO-MDSC in 10ng/mL M-CSF-condition. (**J**) FCM analysis of the proportions of DC after 5 μM DMSO/STM2457 treatment for 5 days of tumor MO-MDSC in 10 ng/mL GM-CSF and 2.5 ng/mL IL-4 condition. (**K**) The mRNA decay curve of Smad3 in tumor MO-MDSC treated with siNC/siMettl3 after Actinomycin D treatment through qRT-PCR data. (**L**) Plasmids diagram containing wild type or mutated versions of m6A sites 3037 A > C and/or 4591 A > C in the Smad3 gene. (**M**) Double luciferase assay analysis of the luciferase activity of five group tumor MO-MDSC transfected corresponding plasmids for 72 hours. (**N**) Double luciferase assay analysis of the luciferase activity of five group tumor MO-MDSC transfected corresponding plasmids for 72 hours with 5 μM DMSO/STM2457. (**O**) RIP-qPCR analysis of the binding of Ythdf2 protein with Smad3 mRNA in tumor MO-MDSC. Except D (n = 5), error bars of statistic graphs represent mean ± SD, n = 3. For **A**, **D**, **M** and **O**, p values were determined using one-way ANOVA with Tukey test; For **B**, **E**, **F**, **I**, **J** and **N**, *p*-values were determined using two sides unpaired t-test. *p < 0.05; **p < 0.01; ****p < 0.0001; ns no significant.

Indeed, m^6^A levels were higher in Smad3 transcripts from BM-MDSC and tumor MO-MDSC than in primary BMC, which showed no notable m^6^A modifications (Fig. 6A). After 48 h of TCCM treatment, m^6^A levels of Smad3 transcripts increased significantly in BMC (Supplementary Fig. 6B). When STM2457, an inhibitor of Mettl3 (the m^6^A methyltransferase), was used during BM-MO-MDSC induction, Smad3 transcripts increased significantly (Fig. 6B) and Smad3 protein levels gradually increased over 72 h (Fig. 6C). Consistently, m^6^A levels in Smad3 mRNA were significantly reduced following Mettl3 knockdown in tumor MO-MDSC (Fig. 6D), accompanied by a notable elevation in Smad3 transcription levels (Fig. 6E), while Mettl3 overexpression decreased Smad3 expression (Fig. 6F). We postulated that the increased m^6^A modification of Smad3 mRNA in MO-MDSC may be attributed to alterations in Mettl3 expression. As expected, Mettl3 expression was significantly higher in BM-MO-MDSC than that in BMC (Fig. 6G). Tumor MO-MDSC also showed higher Mettl3 levels than wild-type CD11b^+^Ly6C^+^ cells, with pMΦ exhibiting the lowest levels (Fig. 6H). After 24 h of ATRA exposure, Mettl3 in tumor MO-MDSC decreased (Supplementary Fig. 6C), similar to the changes seen in ATRA-induced MΦ compared to the original MO-MDSC (Supplementary Fig. 6D). Subsequently determination of Mettl3/m6A’s effect on MO-MDSC differentiation revealed that STM2457 significantly drove its maturation into MΦ and DC (Fig. 6I, J). Therefore, these findings provided evidence that elevated Mettl3 in tumor MO-MDSC led to a decrease in Smad3 expression, which in turn impeding its maturation potential.

mRNA stability serves as a crucial mechanism for the negative regulation of gene transcript levels by m^6^A [39–41]. Indeed, the decay rate of Smad3 mRNA was significant increased from 0.97 hours to 3.04 hours in tumor MO-MDSC after Mettl3 knockdown (Fig. 6K). To further identify the m^6^A sites crucial for Smad3 mRNA stability, a double luciferase assay was performed with plasmids containing wild-type or mutated versions of m^6^A sites (3037 A > C and/or 4591 A > C) in the Smad3 gene (Fig. 6L). It was suggested that the inhibition of Smad3 in tumor MO-MDSC was markedly reversed by mutating the m^6^A site at position 4591, but not 3037 (Fig. 6M), which was restored by administration of STM2457 (Fig. 6N). These results indicate the pivotal role of the m^6^A site located at position 4591 in the stability of Smad3 mRNA.

Considering the preferential recognition of m^6^A sites in the 3’UTR and the negative impact on RNA stability, it was hypothesized that the m^6^A reader Ythdf2 might participate in this regulation of Smad3. This was supported by the strong binding of Smad3 mRNA with Ythdf2, but not Ythdf1/Ythdf3, which is closely dependent on m^6^A modifications (Fig. 6O and Supplementary Fig. 6E). Overall, these findings highlight the importance of Mettl3-mediated m^6^A modification at the 3’UTR of Smad3 mRNA in its low expression in tumor MO-MDSC, due to impaired mRNA stability by Ythdf2 recognition.

### Plasma levels of Tgfβ1 as a predictor of MO-/PMN-MDSC ratio among tumor patients

**Fig. 7:**
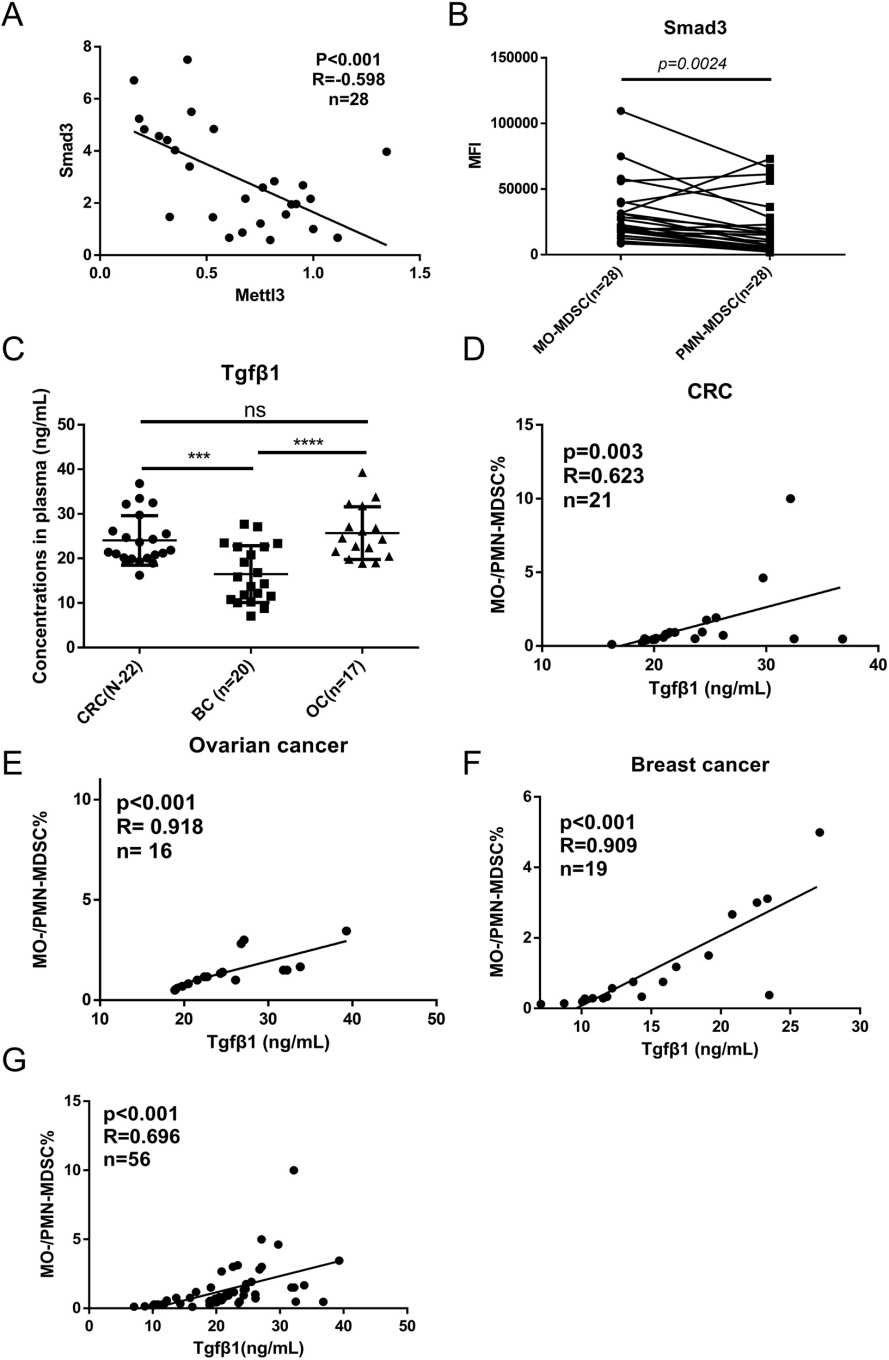
Plasma levels of Tgfβ1 as a predictor of MO-/PMN-MDSC ratio among tumor patients. (**A**) FCM analysis of the correlations between Mettl3 and Smad3 in MO-MDSC derived from CRC patients (n = 28). (**B**) FCM analysis of the Smad3 expressions in CD33^+^CD14^+^CD15^–^HLA-DR^lo^ MO-MDSC and CD33^+^CD14^–^CD15^+^HLA-DR^–^PMN-MDSC derived from CRC patients (n = 28). (**C**) ELISA assays analysis of the concentrations of circulatory Tgfβ1 in the peripheral blood of ovarian cancer (OC), CRC, and breast cancer (BC) patients. (**D**–**F**) Analysis the correlations of plasma Tgfβ1 concentration with MO-/PMN-MDSC ratio in the peripheral blood of CRC, ovarian cancer, and breast cancer patients. (**G**) Analysis the correlations of plasma Tgfβ1 concentration with MO-/PMN-MDSC ratio across CRC, ovarian cancer, and breast cancer patients. For **B**, *p*-values were determined using two sides paired t-test; For **C**, p values were determined using one-way ANOVA with Tukey test; For **A** and **E**–**G**, Spearman’s rank correlation tests were used, R = Spearman rank correlation coefficient. ***p < 0.001; ****p < 0.0001; ns no significant.

Next, we confirmed the expression and correlation of Mettl3 and Smad3 in CRC patients. According to TCGA database, Smad3 expression is significantly lower in CRC patients along with elevated expression of Mettl3 compared to that in healthy controls (Supplemental Fig. 7A, B). Additionally, Smad3 expression was correlated positively with the monocyte maturation marker HLA-DR in CD11b^+^ myeloid cells derived from PBMC of CRC patients, confirming the crucial role of Smad3 in monocytic development (R = 0.625, n = 32) (Supplemental Fig. 7C, Table S2). A significant inverse correlation between Mettl3 and Smad3 expression was also identified in CD33^+^CD14^+^CD15^–^HLA-DR^lo^ MO-MDSC derived from PBMC of patients with CRC (R = -0.598, n = 28) (Fig. 7A, Table S3). Interestingly, Smad3 was found higher levels in MO-MDSC from PBMC of CRC than in PMN-MDSC (Fig. 7B, Table S3). Actually, Smad3 expression is inherently higher in monocytic lineage compared to granulocytic lineage whether in a tumor or normal state (Supplemental Fig. 7D), indicating a preferential role for Smad3 in monocytic development. It was reported that PU.1, a transcription factor that drives monocytic while suppressing granulocytic differentiation, could directly bind with Smad3 in favor of Smad3’s function with genomic regions [42]. A positive regulation was also found of PU.1 in Tgfβ1/Smad3 activation [43]. These findings imply a potential mechanistic interaction between Smad3 and transcription factors in monocytic development and warrant further investigation.

In clinical settings, both MO-MDSC (CD33^+^CD14^+^CD15^–^HLA-DR^lo^) and PMN-MDSC (CD33^+^CD14^–^CD15^+^HLA-DR^–^) had pro-tumor effects, and the ratio of these two subsets was distinct among different tumor types and therefore potentially impacted treatment strategies [44]. Considering the preferential effects of Tgfβ1 in MO-MDSC expansion rather than PMN-MDSC, we tried to explore the potential of plasma Tgfβ1 as a biomarker for distinguishing MO-/PMN-MDSC ratio among tumors. An extensive cohort study by Brandau et al. [44] reported that ovarian cancer patients had a higher relative frequency of MO-MDSC to PMN-MDSC in peripheral blood compared to CRC patients, and breast cancer patients displayed the highest ratio of PMN-/MO-MDSC. Our results showed that plasma Tgfβ1 levels were lower in breast cancer patients than in ovarian cancer and CRC patients, although no significant difference was observed in plasma Tgfβ1 levels between patients with ovarian cancer and CRC (Fig. 7C, Table S4). However, plasma Tgfβ1 levels in all three cancer types exhibited a significant positive correlation with the MO-/PMN-MDSC ratio (Fig. 7D-F). We next further ascertain the predictive potential of Tgfβ1 for MO-/PMN-MDSC ratio across various tumor types. Interestingly, plasma Tgfβ1 levels retained a significant predictive capability across these three tumor types (Fig. 7G). Collectively, these findings indicate a potential utility of plasma Tgfβ1 levels as a predictor for MO-/PMN-MDSC ratio across tumor patients.

## Discussion

MDSC are predominantly characterized by a “two-signal” mechanism [45, 46]. The initial signal usually involves granulocyte-macrophage colony-stimulating factor (GM-CSF), granulocyte colony-stimulating factor (G-CSF), and/or interleukin 1β (IL-1β) [22]-dependent expansion of granule-monocytic precursors (GMP) through the activation of signal transducer and activator of transcription 3 (STAT3) [47, 48], as well as complex regulation of CCAAT-enhancer-binding protein α (C/EBPα) [49], C/EBPβ [50], and interferon regulatory factor 8 (IRF8) [51]. Subsequently, secondary activation is mainly driven by the nuclear factor-κB (NF-κB) pathway, S100A8/A9, and prostaglandin E2 (PGE2) [8]. However, these transcriptional networks during the first signal overlap with broader programs of hematopoiesis in diverse physiological contexts, leading to broad and nonspecific effects on subsequent PMN-MDSC and MO-MDSC differentiation rather than PMN-MDSC or MO-MDSC lineage and cell specificity [52, 53]. Notably, the downregulation of IRF8 observed during the differentiation of MDSC in cancer lesions [54, 55] parallels that observed during the formation of Lin^−^c-Kit^+^Sca-1^−^FcγR^+^ GMP clusters during regenerative myelopoiesis [56]. Moreover, although IRF8-deficient mice exhibited dysregulated myeloid cell differentiation and accumulation of CD11b^+^Gr-1^+^ MDSC, mice with myeloid-specific IRF8 deficiency showed no abnormal myeloid cell lineage differentiation [57]. Furthermore, C/EBPs serve as principal regulators upstream of GMP differentiation, exerting an extensive influence on myeloid specification [58, 59]. Therefore, although these factors have been implicated in the development of MDSC, the precise events governing MDSC lineage commitment remain unclear. The specific mechanism underlying MDSC maturation arrest and the specification of MDSC subsets from hematopoietic precursors is crucial for targeted correction of differentiation and preventing their accumulation in a context-dependent manner.

Our interest was driven by the paradox that, despite abundant Tgfβ1 signaling in tumors, which typically promotes differentiation through Smad2/3, it fails to drive further differentiation of MDSC. In this study, we demonstrate that marked deficiency of Smad3 is critical for maintaining the immature state of tumor MO-MDSC, acting as an underappreciated regulatory factor that impairs MO-MDSC maturation. The importance of Smad3 in monocytic development was first highlighted by the increased frequency of CD11b^+^Ly6C^lo^ cells (with higher CD86 expression) and fewer CD11b^+^Ly6G^+^ granulocytic cells in tumor-free mice overexpressing Smad3 in myeloid cells (Lys2-Smad3-AAV9). Consistently, disrupted differentiation of MΦ and DC was observed in vitro upon Smad3 inactivation. Mice with myeloid-specific overexpression of Smad3 (Lys2-Smad3-AAV9) showed significantly slower tumor progression and enhanced anti-tumor immunity, accompanied by fewer MO-MDSC and more mature MΦ and DC in both tumor tissue and spleen. Notably, direct upregulation of Smad3 expression in tumor MO-MDSC substantially promoted its differentiation into mature MΦ and DC, whereas inactivation of Smad3 blocked this process, confirming the deficiency of Smad3 as a key factor contributing to impaired MO-MDSC maturation. This discovery aligns with prior observations in autoimmune models, where Smad3-deficient mice exhibited a heightened prevalence of NO-producing CD11b^+^Gr1^+^ MDSC [60]. Thus, we identified, for the first time, that transcription factor Smad3 is specific to the differentiation of tumor MO-MDSC but not PMN-MDSC, whose reduction leads to accumulation of immature MO-MDSC during tumor progression.

Intriguingly, Tgfβ1 paradoxically promoted MO-MDSC expansion from BMC, accompanied by increased proliferation capacity as indicated by Ki67 upregulation. This is consistent with prior reports [13, 31]. To resolve this paradox, Smad3 expression was significantly downregulated in TCCM culture, and Smad3 inhibits MO-MDSC differentiation at an early stage in a Tgfβ1-dependent manner, exerting effects throughout the process. Further mechanistic studies revealed the PI3K/AKT pathway, a Tgfβ1 signaling axis [35–37], was significantly activated in MO-MDSC. Pharmacological inhibition of PI3K/AKT abolished MO-MDSC proliferation induced by Smad3 inactivation. In fact, it was observed that PI3K/AKT activation could be enhanced by Smad3 inactivation without alterations in Tgfβ1 receptors (TgfβRII/I). In the present study, Tgfβ1 has not only been reported to promote MO-MDSC expansion, consistent with our findings [13, 61], but also shows the induction of co-stimulatory molecules in MDSC in a Smad2-dependent manner with inhibited iNOS expression, likely indicating MO-MDSC [62]. These findings collectively explain the dual nature of Tgfβ1 in tumors, which may be attributed to context-dependent activation of multiple downstream pathways.

We further explored the molecular mechanisms underlying Smad3 suppression. Strikingly, Mettl3-mediated m^6^A modification destabilized Smad3 mRNA via Ythdf2-dependent recognition of a specific m^6^A site (position 4591) in the 3’UTR, accounting for impaired tumor MDSC maturation.

Mettl3 was highly expressed in MO-MDSC and was responsible for impeding tumor MDSC maturation. Thus, our findings might present a new balance pathway, the degree of m^6^A mRNA modification, for the decision of expansion/differentiation under Tgfβ1 stimulation and a possible explanation for the multifaceted roles of Tgfβ1 in MDSC expansion. Regarding the factors modulating Mettl3 expression in MO-MDSC, it was observed that Mettl3 was significantly promoted by TCCM (data not shown), indicating the possibility of soluble factors in the regulation of Mettl3 expression. In fact, some factors, such as IL-6 and lactic acid, significantly promotion in the expression of Mettl3 in tumor MO-MDSC (data not shown). Interestingly, Tgfβ1 has also been reported to positively regulate Mettl3 expression in lung cancer cells, thereby inducing EMT [63]. These findings highlight a conserved role of tumor microenvironment in suppressing myeloid maturation through Mettl3-mediated epigenetic regulation.

Clinically, plasma Tgfβ1 levels correlated positively with the MO-MDSC/PMN-MDSC ratio across multiple cancer types including CRC, ovarian cancer, and breast cancer, suggesting its potential as a biomarker for stratifying the MO-MDSC/PMN-MDSC ratio in cancer patients.

In summary, our results establish the dual roles of Tgfβ1 in orchestrating MO-MDSC dynamics via the classical Smad3-driven differentiation pathway versus the non-classical PI3K/AKT-mediated proliferative pathway. Furthermore, we revealed Mettl3/m^6^A-dependent RNA decay of Smad3 as the balance mechanism between these two pathways. Additionally, we provided evidence supporting the potential clinical value of Tgfβ1 as a marker of the MO-MDSC to PMN-MDSC ratio among tumor patients.

## Materials & methods

### Animal models

The murine colon cancer cell line CT26 was obtained from Applied Biological Materials Inc. (Zhenjiang, China). CT26 cells were cultured at 37 °C in 5% CO_2_ in RPMI-1640 (Gibco) medium supplemented with 10% heat-inactivated fetal bovine serum (FBS, Gibco, Thermo Fisher Scientific, USA), 100 U/mL penicillin–streptomycin (10,000 U/mL, Gibco), and 50 mM 2-mercaptoethanol (50 mM, Gibco). Cells were split when they were 80% confluent. Cell line was authenticated by STR profiling and mycoplasma negative.

Female mice were maintained on a BALB/c background and analyzed between 6-8 weeks of age. BALB/c mice were purchased from the Animal Experiment Center of Jiangsu University. All mice were maintained in the SPF animal facility at the Animal Experiment Center of Jiangsu University. The mice were subcutaneously injected with 1×10^6^ cells of CT26 cells. Tumor volume was monitored three times per week using the following equation: (length × width^2^)/2. Mice were euthanized with tumors larger than 1.5 cm^3^ and sacrificed according to the experimental requirements.

For mouse myeloid AAV infection, the 6w-old female BALB/c mice with body weights between 18 g and 20 g were selected. Each mouse was anesthetized by subcutaneous injection of 1% sodium pentobarbital (40 mg/kg). After the knee joint skin of the mice was disinfected on an ultra-clean table, 5ul/ AAV at 2 × 10^13 GC/mL concentration containing the Smad3 CDS sequence (AAV9-Lyz2>Kozak-Mouse Smad3 CDS [NM_016769.4]/3×FLAG/P2A/EGFP) or the control sequence (AAV9-Lyz2> Kozak-EGFP) was injected into the right-handed femur of the mice using a microsample. Their weight and growth status were monitored two months after AAV injection.

For all animal experiments, in cases where data differences permitted definitive conclusions, we adhered to the reduction principle of the IACUC guidelines to minimize mouse usage. All animal experiments were randomized and blindly analyzed to minimize experimental bias, in which the investigators were blinded to allocation during experiments and outcome assessment in the statistics part.

### Human PBMC isolation

Peripheral blood samples anticoagulated with K_2_EDTA were collected from healthy controls and tumor patients, maintained at room temperature, and processed for peripheral blood mononuclear cell (PBMC) isolation within 4 h post-collection. The whole blood was centrifuged at 2000 rpm for 10 min at room temperature and cellular pellet was resuspended in an equal volume of pre-warmed PBS. This suspension was gently layered onto 2 mL of room temperature-equilibrated Ficoll-Paque solution (Biosharp, China, BL590) in a sterile conical tube, followed by centrifugation at 2000 rpm for 20 minutes with controlled acceleration and deceleration at room temperature. The buffy coat containing PBMCs was carefully aspirated and transferred into a new tube. The isolated cells were washed once with PBS using centrifugation at 500 g for 5 minutes, and carried out the subsequent flow cytometry staining procedure. All samples were obtained from the Affiliated People’s Hospital of Jiangsu University, and obtained informed consent from subjects. Necessary clinical information of collected samples is provided in Supplemental Tables 1–4 (Tables S1–S4).

### MDSC isolation

MDSC was isolated from the spleens of CT26 mice by magnetic separation (Miltenyi Biotec, Germany) according to the manufacturer’s instructions. CD11b^+^Gr1^+^cells were obtained by staining splenocytes with a purified rat anti-mouse CD11b antibody (BD Pharmingen, USA), followed by positive selection with anti-rat IgG microbeads. The purity was evaluated using flow cytometry.

### Tumor-conditioned medium collection

CT26 cells were seeded at 4 × 10^5^ cells/mL and cultured in RPMI-1640 containing 10% heat-inactivated FBS, 100 U/mL penicillin-streptomycin, and 50 mM 2-mercaptoethanol. 24 hours later, cell-free supernatant passing through 0.2 µm filters was collected, which was called “TCCM” and stored in aliquots at −20 °C subsequently.

### Induction of BM-MDSC

BM cells were obtained from hind limb bones (femurs, tibias), harvested immediately after euthanasia, and stored in cold RPMI-1640 culture buffer (10% FBS, 1% penicillin-streptomycin) under sterile conditions. Bones were crushed using a 1 mL syringe, then passed through a 40 μm cell strainer to obtain single-cell suspensions for downstream applications. After blood cell lysis, BM cells were cultured for 4 days, supplemented with 20 ng/mL of IL-6 (Peprotech) and GM-CSF (Peprotech), or in the presence of 50% TCCM with 20 ng/mL GM-CSF in 1640 complete culture buffer. In a study on the regulation of Tgfβ, Smad3, or PI3K/AKT in MDSC differentiation, Tgfβ 10/20/50 ng/mL (Peprotech, 100-21), Anti-Tgfβ antibody 10 μM (Selleck, A2113), IgG1 isotype control 10 μM (Selleck, A2119), SIS3 10 μM (MCE, HY-13013), Wortmannin 2.5 μM (MCE, S2758) were added to each group. The cells were cultured in RPMI-1640 medium containing 10% heat-inactivated FBS, 100 U/mL penicillin–streptomycin, and 50 mM 2-mercaptoethanol in a 37 °C incubator with 5% CO_2_.

### Induction of MΦ and DC

For induction of MΦ from BM cells, 3 × 10^5^ BM cells were plated in 96-well plates per well and treated with M-CSF (20 ng/mL; Peprotech, 100-21) for 7 days. Change the solution every three days. For induction of MΦ from MDSC, only M-CSF 2500 U/mL (Peprotech, 100-21) or ATRA 2 μM (Sigma, R2625) with 20% TCCM and 10 ng/mL GM-CSF were added for 5 days according to previous reports [64].

GM-CSF (20 ng/mL; Peprotech, 315-03-20) and IL-4 (10 ng/mL; Peprotech, 214-14-20) were added to the BM cell culture. Three days later, the medium was replaced with 3/4 of the culture medium and supplemented with cytokines. After culturing for 3 days, the cells were collected, re-laid on plates, and replenished with cytokines. Cells were collected on the 8th day of BM cell culture for morphological and purity identification. For the induction of DC from MDSC, 10 ng/mL GM-CSF and 2.5 ng/mL IL-4 were added to the collected MDSC continually cultured for 5 days in a frequency of 1/2 medium change for 3 days, and the underlying adherent cells were collected for the following experimental requirements. The cells were cultured in RPMI-1640 medium containing 10% heat-inactivated FBS, 100 U/mL penicillin–streptomycin, and 50 mM 2-mercaptoethanol in a 37 °C incubator with 5% CO_2_.

### Peritoneal macrophages (PMΦ) isolation

After the mice were disinfected, 10 mL PBS was gently injected into the abdominal cavity with syringes, and the abdomen was gently massaged to evenly mix the cells in the abdominal cavity. The mixture was collected in the abdominal cavity and centrifuged for 500 g, 5 min to precipitate the cells. The cells were cultured in 6-well plates and RPMI-1640 medium containing 10% heat-inactivated FBS, 100 U/mL penicillin–streptomycin, and 50 mM 2-mercaptoethanol at 37 °C under 5% CO_2_ for 3 h, and adherent peritoneal macrophages were collected.

### siMettl3 transfection and STM2457 treatment

The Small interfering RNAs against Mettl3 (siMettl3) and the negative control (siNC) were designed and synthesized by GenMute (Suzhou, China). For tumor MO-MDSC transfection, cells were seeded in 24-well plates at appropriate densities to achieve a confluence of 80% to 90% at the time of transfection; Cells were transfected with siRNA using Lipofectamine 3000 (Invitrogen, CA, USA) according to the manufacturer’s instructions and refreshed the medium 6 hours after transfection. The efficiency of transfection was validated using qRT-PCR. The interfering sequences were listed in the online Supplemental Table 5 (Table S5).

For inhibiting the enzyme activity of Mettl3, 5 μM STM2457 (MCE, HY-134836) or DMSO as control was used in 24-well plate with 2 × 10^6^ MDSC per well and collecting cells according to experiment requirements. Cells were cultured in a 37 °C incubator with 5% CO_2_.

### CFSE-labeling assay

Round-bottom 96-well plates were coated overnight at 4 °C with 50 μL per well of anti-CD3 monoclonal antibody at a final concentration of 1 μg/mL. CD8⁺ T cells were isolated from wild-type mouse spleens using immunomagnetic sorting kit (Stem Cell Technologies, 19858) and resuspended in pre-warmed PBS containing 1% BSA at a density of 1 × 10⁷ cells/mL. CFSE dye (Sigma, 150347-59-4) was added to a final concentration of 3 μmol/L, followed by incubation at 37 °C for 10 min under dark. The reaction was stopped with 5 volumes of ice-cold RPMI-1640 medium supplemented with 10% FBS. Cells were centrifuged at 500 g for 5 min at 4 °C and washed three times with RPMI-1640 containing 10% FBS. CFSE-labeled CD8⁺ T cells were co-cultured with freshly isolated or cultured MDSC or MO-MDSC at a 1:1 ratio in the pre-coated plates within complete RPMI-1640 medium containing anti-CD28 mAb at a final concentration of 1 μg/mL. After 72 h, cells were harvested and labeled with 0.25 μg APC-anti-CD8 antibody in 100 μL PBS at 4 °C for 30 min. Flow cytometry analysis was conducted to assess the results.

### Lentiviral transfection

The mouse full-length Smad3(NM_016769) coding sequence was introduced into the BamHI/AgeI of GV492 Vector (GENECHEM, Shanghai, China) and transfected with lentiviruses. The virus transfection agents polybrene and lentivirus were introduced according to the manufacturer’s instructions in a suspension of 2 × 10^6^/mL MDSC. The mixture was then thoroughly mixed and centrifugation at 150 g for 1 h at room temperature. Following centrifugation, the culture was maintained at 37 °C under 5% CO_2_ for a period of 2–5 days as required by the experiment, with the liquid being changed as necessary based on cell growth. Flow cytometry and Western blot were used to detect the overexpression efficiency.

### Arg-1 activity

Cells were harvested and subjected to lysis using RIPA buffer to facilitate protein extraction. The subsequent detection process was conducted following the manufacturer’s protocol. The absorbance of the reaction wells was measured at a wavelength of 520 nm. The enzymatic activity of Arginase-1 (Arg-1) was determined using the formula: Arg-1 Activity = (A _Sample_ - A _Sample Blank_) / (A _Standard_ - A _Standard Blank_). A, absorbance.

### NO concentration

Cell culture supernatants were collected. Operate according to the Nitric Oxide Synthase (NOS) Assay Kit (Sigma, USA, MAK532) and draw a standard curve by Curve Expert 1.4 software. The absorbance of each well was measured at a wavelength of 520 nm, and the concentration of nitric oxide (NO) was calculated according to the standard curve.

### ROS detection

One to five million cells were collected, resuspended in 1 mL PBS supplemented with 3 μL PMA (10 μg/mL, Sigma, USA, P1585) and 0.5 μL H₂DCFDA (2.5 μM, Thermo Fisher Scientific, USA, D399), and incubated protected from light at 37 °C for 1 h. After centrifugation at 500 g for 5 min at 4 °C, the pellet was washed twice with PBS. Finally, cells were resuspended in 200 μL PBS and subjected to ROS fluorescence intensity analysis by flow cytometry.

### Cell fluorescent labeling of MDSC

Preparation of 2 μM Cell Tracker Red CMTPX Dye (Invitrogen, C34552) working solution. MDSC was suspended at a concentration of 5 × 10^6^ cells/mL by preheating the CMTPX working solution at 37 °C and placed in a water bath at 37 °C for 15 min. MDSC were homogenized every 5 min. Centrifugation to remove excess CMTPX dye solution. Early CT26 tumor-bearing mice (tumor volume of approximately 0.8 cm^3^) were intratumorally injected with PBS at a concentration of 5 × 10^6^ cells/200 μL. After 3–5 days, the phenotype and distribution of MDSC were detected by flow cytometry according to the experimental requirements.

### mRNA stability assay

The transcriptional inhibitor, Actinomycin D (2 μM, Selleck Chemicals, TX, USA, 50-76-0), was used to suppress mRNA transcription. Subsequently, samples were collected at 0, 2, and 4 h post-treatment with actinomycin D. Total RNA extraction was performed using an RNA-Quick Purification Kit (Shanghai Yishan Biotechnology Co., Ltd ES-RN001). The HPRT1 housekeeping gene was used as the reference for normalization (Sangon Biotech, China). Notably, HPRT1 mRNA lacks m^6^A modifications, remains unbound by Ythdf2, and exhibits minimal susceptibility to the effects of Actinomycin D treatment [65, 66].

### Flow cytometry (FCM) analysis

One to five million cells were resuspended in PBS, followed by incubation with a fluorescent-conjugated antibody. For cell surface marker staining, cells were resuspended in PBS at 1×10⁷ cells/mL, and 100 µL of the suspension was transferred to a 1.5 mL microcentrifuge tube. After adding 0.5 µg of fluorochrome-conjugated primary antibody, the cells were incubated for 30 min at 4 °C with gentle shaking, washed once with 1 mL PBS, and finally resuspended in 200 µL PBS for analysis.

For protein staining involved in nuclear such as Ki67, Foxp3, and Smad3, the pellet from surface-stained cells was treated with nuclear permeabilization buffer (Invitrogen, USA, 88-8824-00) and incubated for 30 min at 4 °C with gentle shaking, followed by one wash as manufacturer’s instructions. For Ki67 and Foxp3 staining, 0.5 µg of fluorochrome-conjugated primary antibody was added and incubated for 1.5 h at 4 °C with gentle shaking, after which cells were washed twice and resuspended in 200 µL buffer. For Smad3 staining, cells were incubated with 0.5 µg rabbit anti-mouse Smad3 primary antibody for 1.5 h at 4 °C, washed once, and resuspended in 50 µL buffer. Then, 0.5 µL AF488-conjugated goat anti-rabbit IgG mAb was added and incubated for another 1.5 h at 4 °C with gentle shaking, followed by two washes and resuspension in 200 µL buffer.

For detection of cytoplasmic or transmembrane proteins such as Granzyme B, CD206, Arg-1 and iNOS, the surface-stained cell pellet was permeabilized using membrane permeabilization buffer with incubation for 30 min at 4 °C under gentle shaking and washed once. Then, 0.5 µg fluorochrome-conjugated primary antibody was added and incubated for 45 min at 4 °C with gentle shaking, after which the cells were washed twice and resuspended in 200 µL buffer for analysis.

Flow cytometry (FCM) was operated using the Beckman Coulter System. The software program used for data analysis was FlowJo, version 10.8.1. The antibodies and fluorescent labels used in this study were as follows: CD11b (PECY7, Biolegend, 101216), Gr-1 (PE, Biolegend, 108408), Ly6G (PE, Biolegend, 127608), Ly6C (APC, Biolegend, 128016), Ki67 (ef450, Invitrogen, 48-5699-42), CD11c (PE, Biolegend, 117308), MHC II (ef450, Invitrogen, 48-5321-82), F4/80 (PE, Biolegend, 11604), CD206 (APC, Biolegend, 141708), Arg-1 (PE, Biolegend, 165804), iNOS (APC, Biolegend, 696808), CD8 (APC, Biolegend, 100712), CD4 (PECY7, Biolegend, 100422), Granzyme B (PE, Biolegend, 372208), CD25 (PECy5.5, Invitrogen, 45-0251-82), and Foxp3 (PE, Biolegend, 126404). Murine MDSC, CD11b^+^Gr-1^+^; murine PMN-MDSC, CD11b^+^Ly6G^hi^; murine MO-MDSC, CD11b^+^Ly6C^hi^; macrophages, CD11b^+^F4/80^+^Ly6C^low^MHCII^+/-^; M1 macrophage, CD11b^+^F4/80^+^ MHCII^hi^CD206^-^; M2 macrophage, CD11b^+^F4/80^+^CD206^+^MHCII^-/lo^; DC, CD11c^+^MHCII^+^; Cytotoxic T lymphocyte (CTL), CD8^+^Granzyme B^+^; Treg, CD4^+^ CD25^+^ Foxp3^+^. Human MO-MDSC, CD33^+^CD11b^+^HLADR^-^CD14^+^CD15^-^; Human PMN-MDSC, CD33^+^CD11b^+^HLADR^-^CD14^-^CD15^+^.

### RIP-qPCR

The cells were collected, and the Magna RIP™ RNA-Binding Protein Immunoprecipitation Kit (Sigma Catalog No. 17-700) was used for the RIP assay. Lysate cells in the RIP lysate buffer and Pre-use Magnetic Beads Protein A/G Sigma CS203178 against the target protein of interest (Mettl3 (ab195352), Ythdf1 (Proteintech 17479-1-AP), Ythdf2 (Abcam Ab246514), and Ythdf3 (Proteintech 25537-1-AP)) were combined for 4 h. The corresponding rabbit or Mouse IgG antibody (Normal Mouse IgG Sigma CS200621; Rabbit IgG Purified Sigma PP64B) was used as a negative control. The magnetic bead-protein antibody complex was adsorbed using a miniature magnetic frame, and the unbound substance was fully washed away. The magnetic bead-protein antibody complex was incubated with the cell lysate overnight, and 10% of the cell lysate was used as the input. The unbound compounds were removed from the micromagnetic rack, and after full washing, mRNA was extracted using the Trizol method together with the input samples. Random primers were used for reverse transcription amplification. Specific primers were used for qRT-PCR to detect the mRNA expression level of the target genes, and the difference in the content of the target gene binding to the target protein in different treatment groups was further analyzed and compared.

### MeRIP-qPCR

Total mRNA was extracted from cultured or sorted cells by the column extraction method (1–20 μg), The EpiQuik™ CUT&RUN m6A RNA Enrichment (MeRIP) Kit (EpiGentek P-9018) containing all the reagents required to carry out successful m6A RNA enrichment starting from total RNA. In the reaction, RNA sequences at both ends of the target m6A-containing regions were cleaved/removed, and the target m6A-containing fragments were pulled down using a bead-bound m6A capture antibody, non-immune IgG control, and m6A positive control. The enriched RNA was released, purified, and eluted. 200–400 ng mRNA was divided into other parts as input. The eluted and input RNA were amplified by reverse transcription using random primers. The specially designed primers (Smad3 forward primer 5’-3’ ATTCCATTCCCGAGAACACTAA Reverse primer 3’-5’ TAGGTCCAAGTTATT GTGTGCT; Tgfβ1 forward primer 5’-3’ CCAGATCCTGTCCAAACTAAGG Reverse primer 3’-5’ CTCTTTAGCATAGTAGTCCGCT) were used for specific amplification of the transcribed cDNA, and the mRNA expression level of the target gene was detected by qRT-PCR to further analyze and compare the differences in the m6A levels of target genes in different treatment groups.

### Double luciferase reporting assay

The firefly luciferase reporter gene recombinant plasmid H306 of Smad3 (NM_016769.4) CDS + 3’UTR WT/Mut was prepared using the pPMIR-Report Luciferase plasmid (OBIO Scientific Service). The lipo3000 transfection reagent was used to co-transfect the firefly luciferase reporter gene plasmid and sea kidney luciferase internal reference plasmid into MDSC cells. The cells were then treated with either 5 μM DMSO/STM2457 or left untreated. After 72 h, luciferase intensity was measured following the instructions provided by the double luciferase reporter gene detection kit (Promega E1910) and the luciferase detection instrument. The relative luciferase activity among the different treatment groups was determined. The wild-type and mutant luciferase plasmids were as follows: pMIR-REPORT Luciferase-Smad3 3’UTR, pMIR-REPORT Luciferase-Smad3 3’UTR (MUT 3037 A > C), pMIR-REPORT Luciferase-Smad3 3’UTR (MUT 4591 A > C), pMIR-REPORT Luciferase-Smad3 3’UTR(MUT3037A > C,4591 A > C).

### ELISA

To obtain human plasma, peripheral blood samples containing anticoagulants were collected and subjected to centrifugation at 2000rpm for a duration of 10 min at room temperature. The resulting supernatant was collected.

To obtain cell culture supernatants, equal numbers of MDSC or tumor cells were seeded and cultured in RPMI-1640 (Gibco, USA) complete medium at a temperature of 37 °C and a CO_2_ concentration of 5%. After 24 h, the culture media were collected and centrifuged at 500 g for 5 min to eliminate any precipitates. The levels of Tgfβ1 were measured using commercially available ELISA kits (Multi Science, EK981, USA).

### Western blot analysis

Total proteins from the cells were extracted by RIPA lysis buffer (Cowin Biotech, China) containing 1% phosphatase and protease inhibitor cocktail (UU-Bio Technology, China). Equivalent amounts of protein samples were separated by 10% SDS-PAGE, with electrophoresis performed at 80 V in the stacking gel and 110 V in the resolving gel (Bio-Rad). Subsequently, proteins were transferred onto a 0.22 μm PVDF membrane in an ice-cold transfer buffer at 350 mA for 1.5 h using a sandwich assembly. The membranes were blocked with 5% non-fat milk (for non-phosphorylated protein) or BSA (for phosphorylated protein) for 1 h at room temperature and then incubated with primary antibodies overnight at 4 °C. After washing three times with TBST (containing 0.5% Tween-20) for 10 min each, the membranes were probed with corresponding secondary antibodies for 1 h at room temperature. Following additional washes, protein bands were visualized using the e-BLOT Touch Imager S system. Details regarding all antibodies are provided online Supplemental Table 6 (Table S6). Full and uncropped western blots are provided online Supplemental Material 2.

### Real-time fluorescence quantification PCR

RNA-Quick Purification Kit (esunbio, China, ES-RN001) was used to extract and purify total RNA. cDNA library was synthesized using 5× All-In-One RT MasterMix (Vazyme, China). Gene expression was quantified with qPCR using target-specific primers (Sangon Biotech, China) and ChamQ SYBR Master Mix (Q511-02, Vazyme). Reactions were performed on a Real-Time PCR System (Jim-X, Bio-Rad). The relative gene expression level was obtained using the 2^−ΔΔCt^ method after normalization to β-actin (loading control). All the primers used for qRT-PCR are listed in Supplementary Table 7 (Table S7).

### Statistical analysis

Statistical analysis and graphing were performed using GraphPad Prism software (version 9). Normality of distribution was assessed using the Shapiro-Wilk test by SPSS software (IBM SPSS Statistics 27.0.1). The homogeneity of variance was assessed by the F test (two groups) or Brown-Forsythe test ( ≥ 3 groups) (GraphPad Prism software (version 9)), and all variance is similar between the groups that are being statistically compared. Two-tailed Student’s t-test, one-way analysis of variance (ANOVA) with Tukey’s test, and Spearman correlation analysis were performed for statistical comparisons. PASS software was used to predetermine the sample size for in-vitro assays to meet the effect size of 0.8 and the α value of 0.05, and our sample sizes are similar to those reported in previous publications [67]. For animal experiments, we adhered to the reduction principle of the IACUC guidelines to minimize mouse usage. All experiments were repeated a minimum of three times with independent biological or technical replicates. No animals or data points were excluded from the analysis. Data are presented as mean ± standard deviation (SD). A two-tailed p value of less than 0.05 was considered statistically significant for all figures (*p < 0.05; **p < 0.01; ***p < 0.001; ****p < 0.0001; ns no significant).

## Supplementary information

Supplementary figures

Supplementary figure legends

Supplementary tables

Supplemental Material 1-cell line authentication

Supplemental Material 2-Primary Western blot picture

## Data Availability

All data generated or analyzed during this study are included in the published article. The data sets used and/or analyzed in this study are available upon request from the corresponding author.
