# Supplementary material for: Downregulated Smad3 signaling impairs the maturation of MO-MDSC in colorectal cancer
Source: Cell Death Dis. 2025 Dec 8;16(1):880. doi: 10.1038/s41419-025-08228-1 (PMC12686534; doi:10.1038/s41419-025-08228-1)
Supplement: Supplementary file 1 — Supplementary figures [file 41419_2025_8228_MOESM1_ESM.docx]

**Supplementary Fig. 1.**


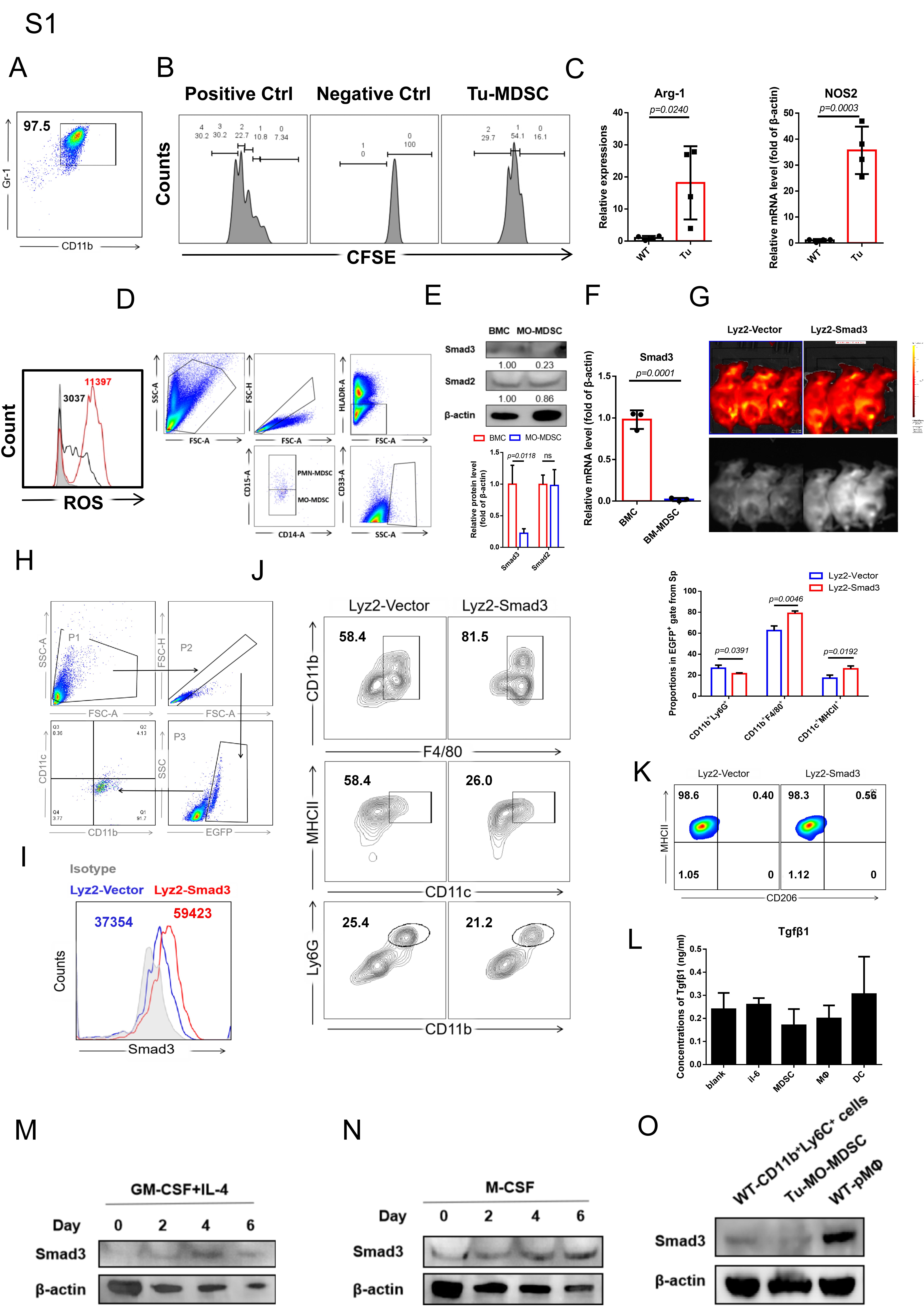


**Supplementary Fig. 2.**


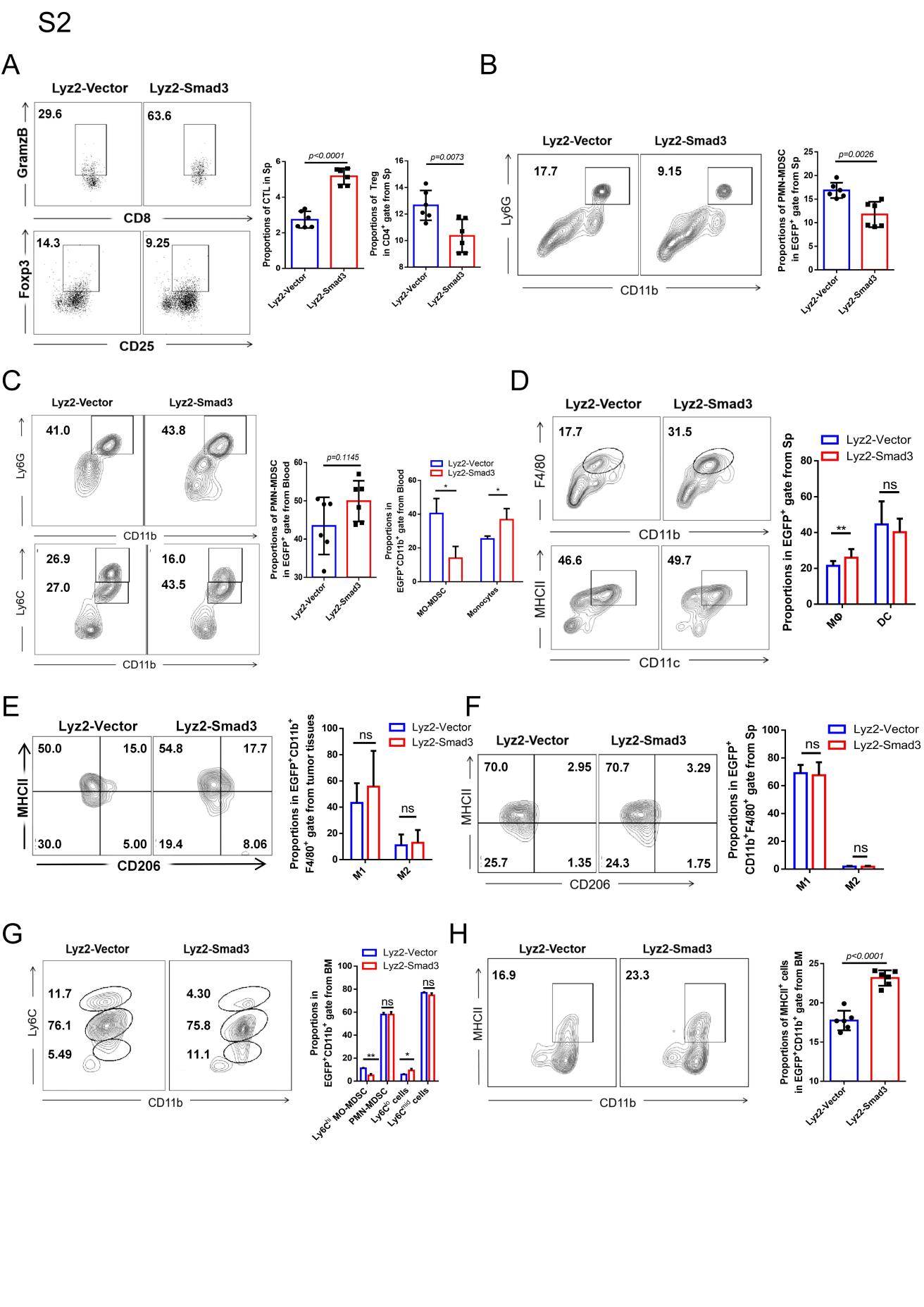


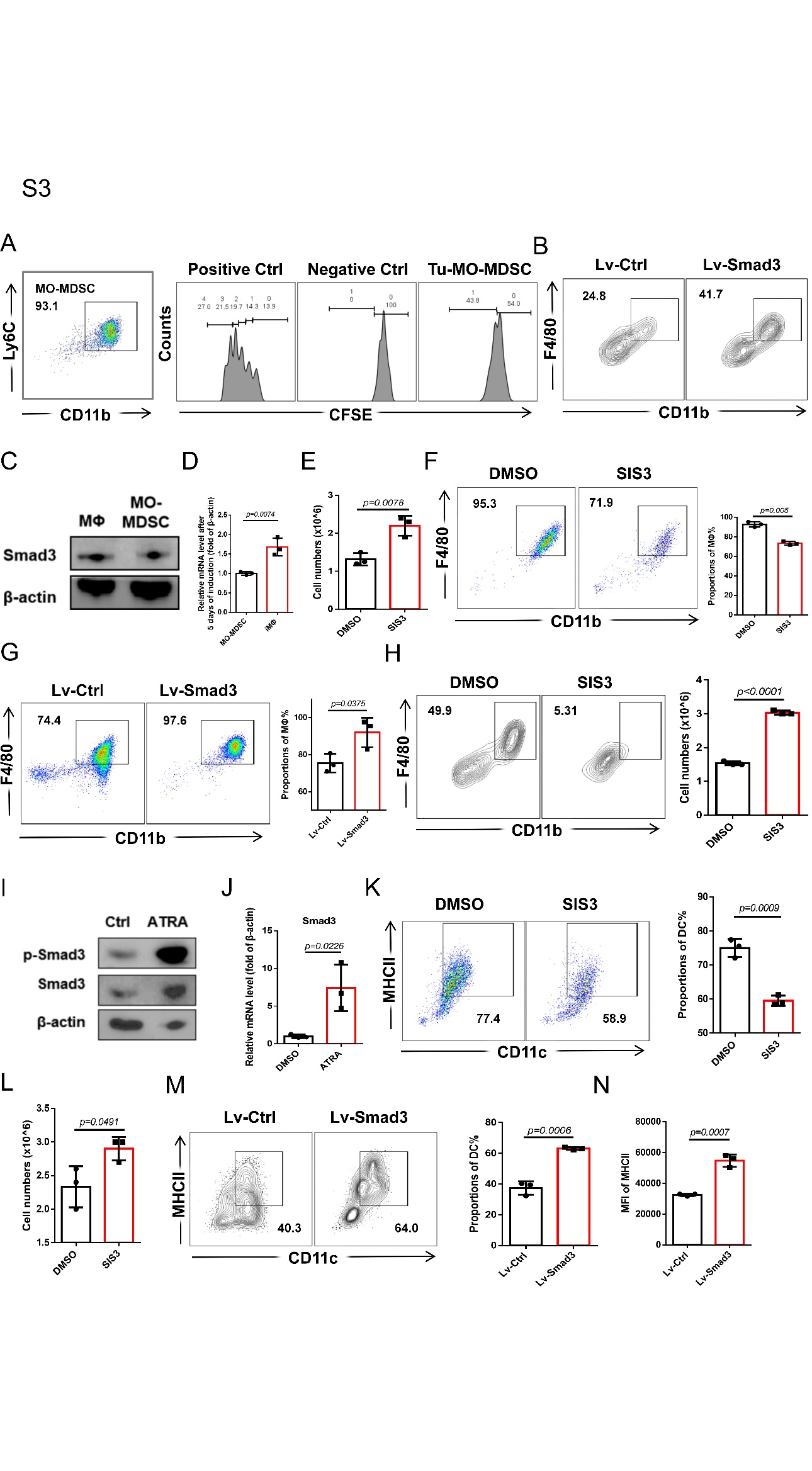
**Supplementary Fig. 3.**


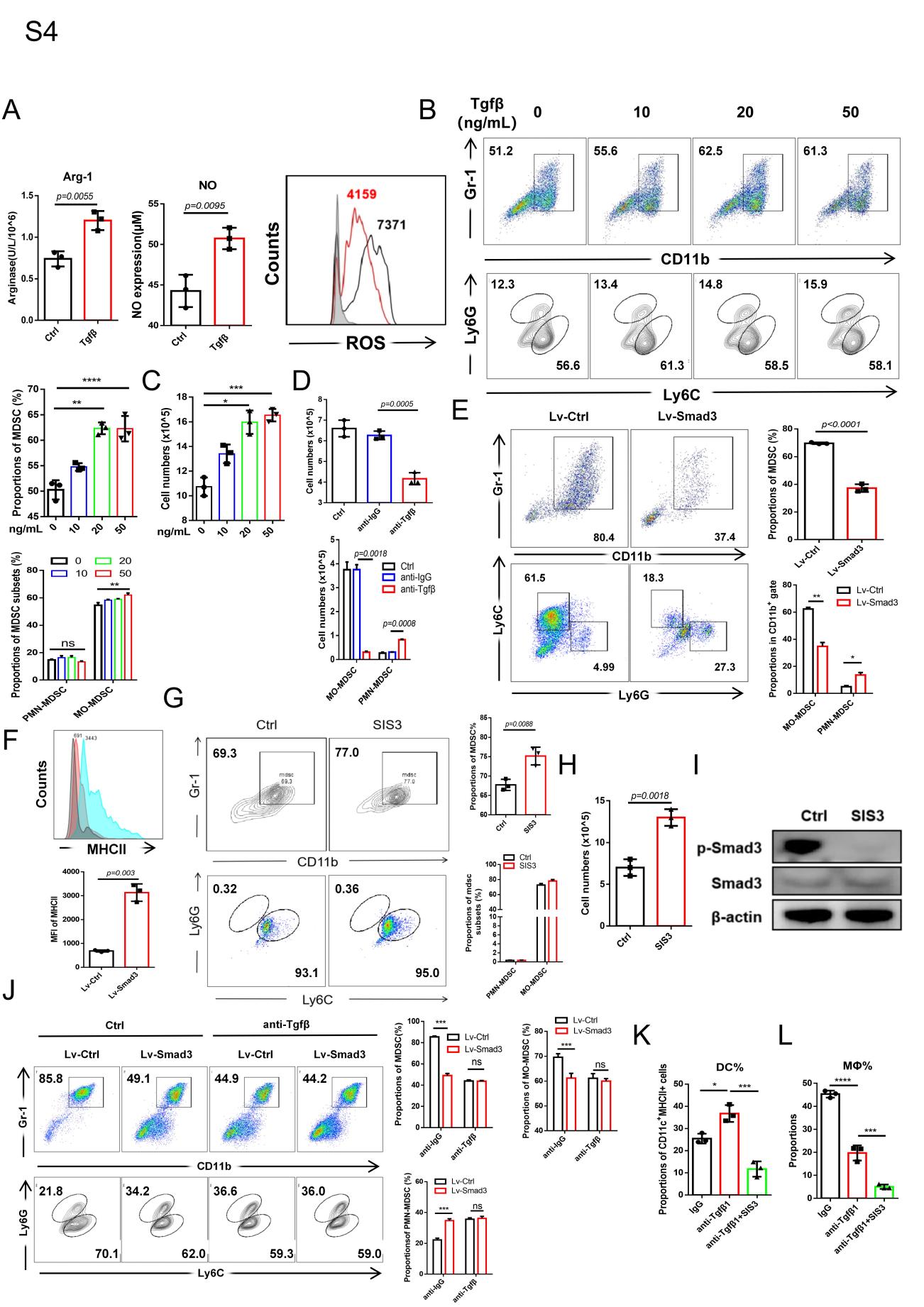
**Supplementary Fig. 4.**


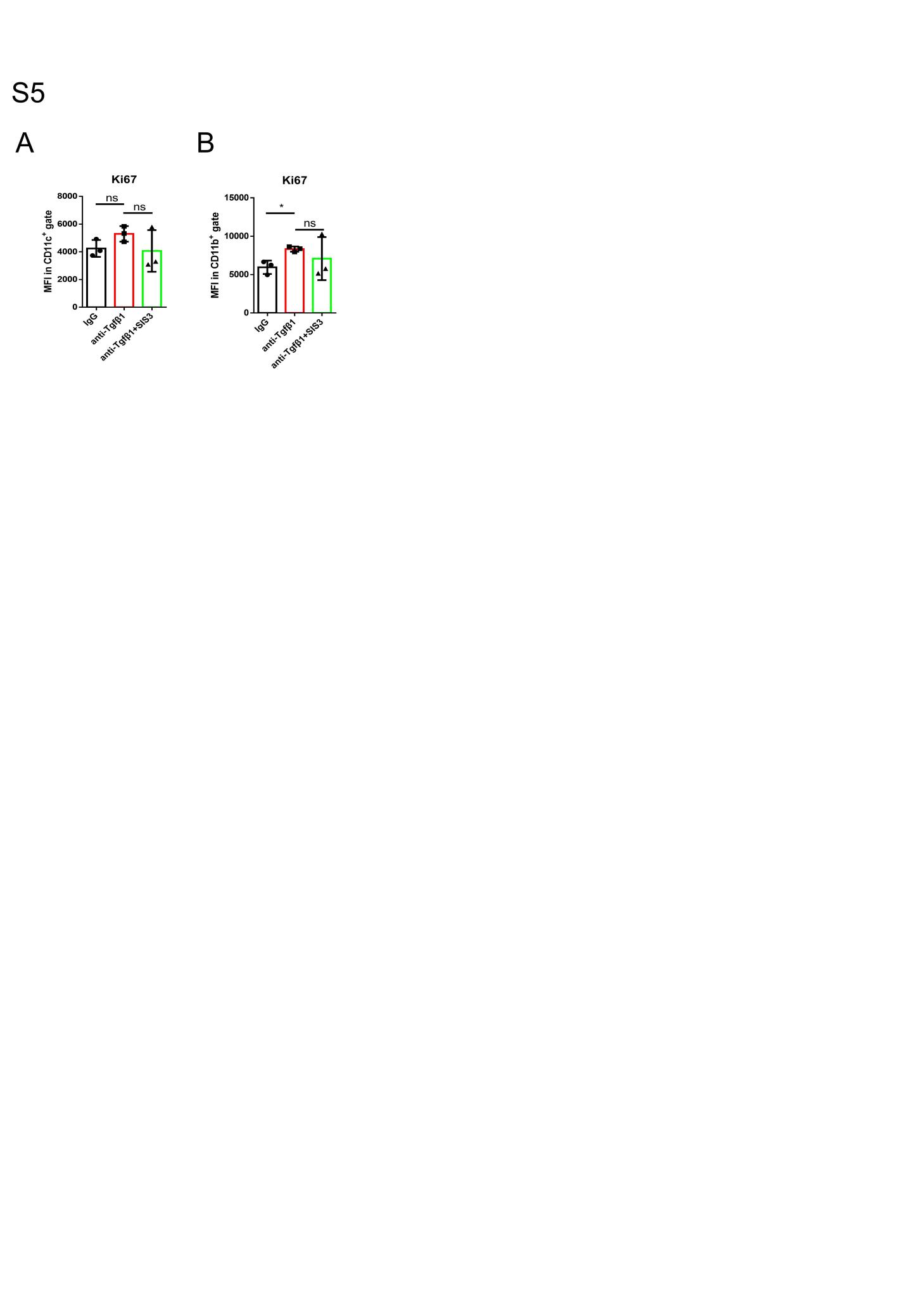
**Supplementary Fig. 5.**


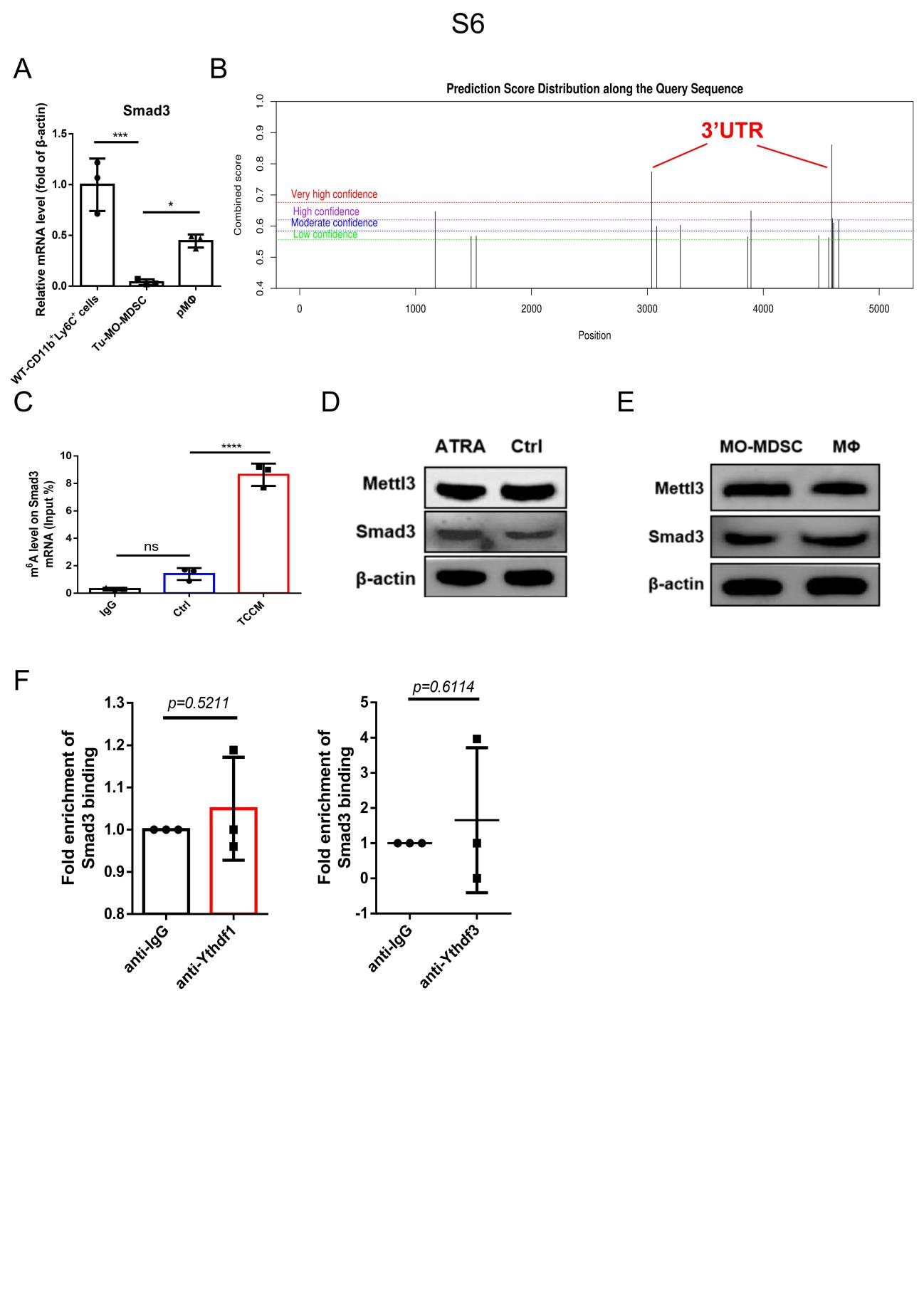
**Supplementary Fig. 6.**


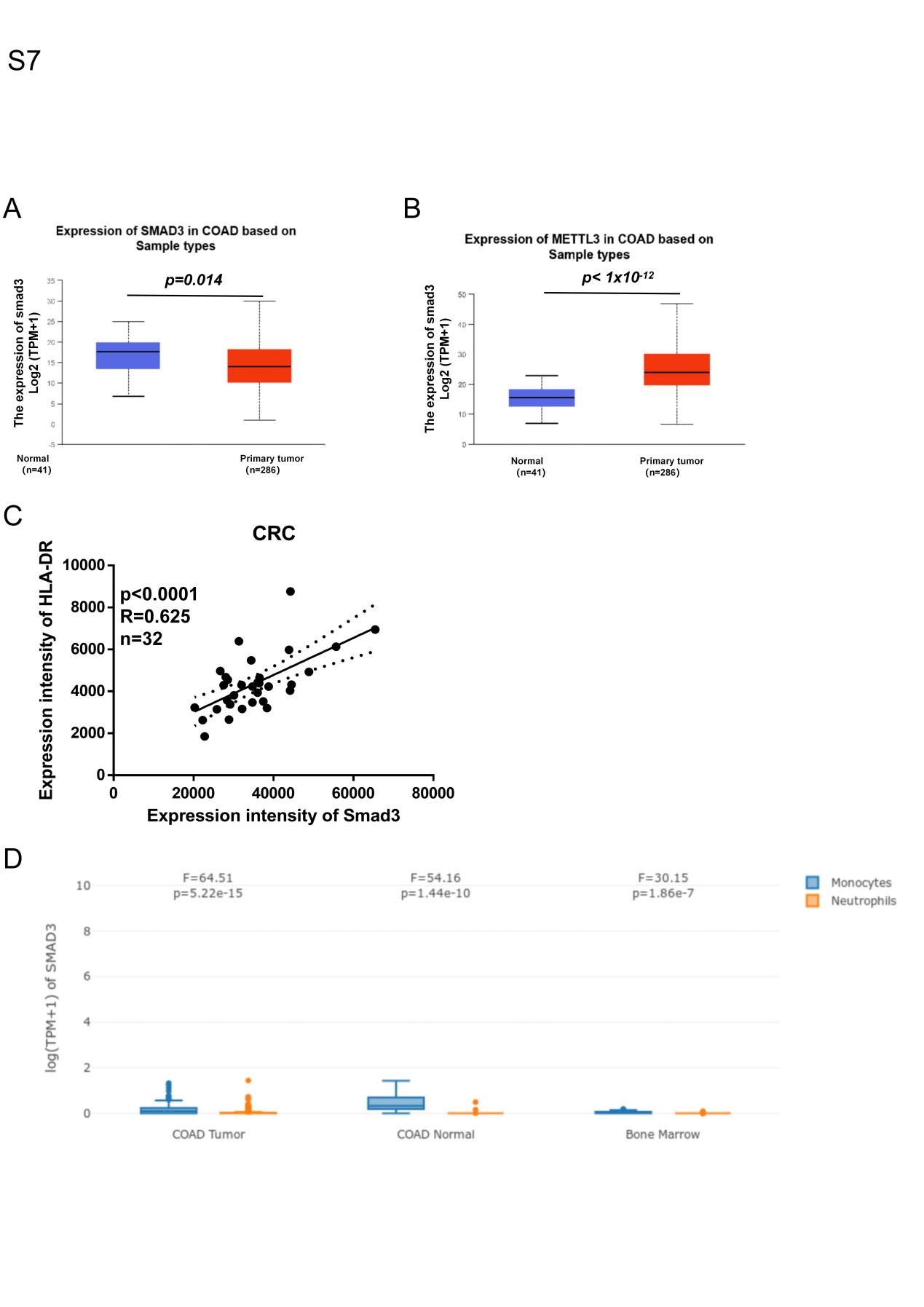
**Supplementary Fig. 7.**
