# Supplementary material for: Downregulated Smad3 signaling impairs the maturation of MO-MDSC in colorectal cancer
Source: Cell Death Dis. 2025 Dec 8;16(1):880. doi: 10.1038/s41419-025-08228-1 (PMC12686534; doi:10.1038/s41419-025-08228-1)
Supplement: Supplementary file 2 — Supplementary figure legends [file 41419_2025_8228_MOESM2_ESM.docx]

**Supplementary Fig. 1.**

(A) FCM identifies the purity of isolated MDSC from the spleen of CT26-bearing mice. (B) Detection of immunosuppressive functions of tumor-bearing MDSC to CD8^+^T cell proliferation isolated from naïve mice via CFSE-labeling experiment. (C) qRT-PCR analysis of the transcription levels of Arg-1, iNOS and FCM analysis of ROS expressions in CT26-derived MDSC and wide-type mice-derived CD11b^+^Ly6G^hi^ cells. (D) Gate strategy of CD33^+^ HLA-DR^lo^ MDSC, CD33^+^CD14^+^CD15^–^HLA-DR^lo^ MO-MDSC and CD33^+^CD14^-^CD15^+^HLA-DR^lo^ PMN-MDSC from PBMC of CRC patients. (E) Western blot analysis of the expressions of Smad2/3 in BMC and the TCCM-induced BM-MDSC. (F) qRT-PCR analysis of the transcription level of smad3 in BMC and the TCCM-induced BM-MDSC. (G) Animal imaging identifies the successfully expressed myeloid smad3 in the limbs of mice from both the AAV9-Smad3 group and the control group after 4 weeks through EGFP fluorescence. (H) Gate strategy of EGFP^+^CD11b^+^ cells in the bone marrow of AAV9-Smad3/vector group of mice. (I) FCM analysis of the expressions of Smad3 in the EGFP^+^CD11b^+^ cells of bone marrow from AAV9-Smad3/vector group of mice. (J) FCM analysis of the proportions of CD11b^+^F4/80^hi^, CD11c^+^MHCII^+^ and CD11b^+^Ly6G^hi^ cells in the EGFP^+^ cells of spleen from AAV9-Smad3/vector group of mice. (K) FCM analysis of the proportions of CD206^+^MHCII^lo^ M2 and CD206^-^MHCII^+^ M1 in the EGFP^+^CD11b^+^F4/80^hi^ cells of spleen from AAV9-Smad3/vector group of mice. (L) ELISA analysis of the secreted Tgfβ1 concentrations in cultural mediums of Ctrl/ 20 ng/mL IL-6 treated BMC for 3 days, GM-CSF and IL-6-induced MDSC for 3 days, 10 ng/mL M-CSF induced MΦ for 7 days and 20 ng/mL GM-CSF adding 10 ng/mL IL-4 induced DC for 9 days. (M, N) Western blot analysis of the expressions of Smad3 during MΦ and DC inductions. (O) Western blot analysis of the Smad3 expressions in wide-type mice-derived CD11b^+^Ly6C^+^ cells (WT-CD11b^+^Ly6C^+^), CT26-isolated spleen CD11b^+^Ly6C ^+^ MDSC (Tu-MO-MDSC) and wide-type mice-derived peritoneal macrophages (pMΦ). Error bars of statistic graphs represent mean ± SD from 3 independent experiments (C, E, F, J, K). For C, E, F and J, p values were determined using two sides unpaired t-test. ns, no significant.

**Supplementary Fig. 2.**

(A) FCM analysis of the proportions of CD8^+^Granzyme B^+^ CTL and CD4^+^CD25^+^Foxp3^+^ Treg cells within spleen of Lyz2-Smad3/vector group of mice bearing CT26. (B) FCM analysis of the proportions of CD11b^+^Ly6G^hi^ PMN-MDSC within spleen of Lyz2-Smad3/vector group of mice bearing CT26. (C) FCM analysis of the proportions of CD11b^+^Ly6C^hi^ MO-MDSC, CD11b^+^Ly6G^hi^ PMN-MDSC within peripheral blood of Lyz2-Smad3/vector group of mice bearing CT26. (D) FCM analysis of the proportions of CD11b^+^F4/80^hi^ MΦ and CD11c^+^MHCII^+^ DC within spleen of Lyz2--Smad3/vector group of mice bearing CT26. (E) FCM analysis of the proportions of CD206^+^MHCII^lo^ M2 and CD206^-^MHCII^hi^ M1 within EGFP^+^CD11b^+^F4/80^+^ cells in tumor tissues in Lyz2-Smad3/Vector group mice. (F) FCM analysis of the proportions of CD206^+^MHCII^lo^ M2 and CD206^-^MHCII^+^ M1 in the EGFP^+^CD11b^+^F4/80^hi^ MΦ of spleen from Lyz2--Smad3/vector group of mice bearing CT26. (G) FCM analysis of the proportions of CD11b^+^Ly6G^hi^ PMN-MDSC, CD11b^+^Ly6C^hi^MO-MDSC, CD11b^+^Ly6C^mid^, and CD11b^+^Ly6C^lo^ cells in the EGFP^+^CD11b^+^ cells of bone marrow from Lyz2-Smad3/vector group of mice bearing CT26. (H) FCM analysis of the expressions of MHCII in the EGFP^+^CD11b^+^ cells of bone marrow from Lyz2-Smad3/vector group of mice bearing CT26 tumor. Error bars of statistic graph represent mean ± SD, n=6. p values were determined using two sides unpaired t-test. *,p< 0.05; **,p< 0.01; ns, no significant.

**Supplementary Fig. 3.**

(A) The purity and immunosuppressive functions to CD8^+^T cell proliferations of CD11b^+^Ly6C^+^ MO-MDSC isolated from CT26-bearing mice. (B) FCM analysis of the proportions of CD11b^+^F4/80^+^ MΦ maturated from tumor MO-MDSC treated with lentivirus-transfected Smad3/Ctrl for 5 days under TCCM condition. (C, D) Western blot and qRT-PCR analysis of the Smad3 expressions in tumor MO-MDSC and the M-CSF-induced MΦ (iMΦ). (E, F) Cell numbers and FCM analysis of the proportions of CD11b^+^F4/80^+^ MΦ maturated from bone marrow-induced MO-MDSC treated with 2μM DMSO/SIS3 for5 days under 10ng/mL M-CSF condition. (G) FCM analysis of the proportions of CD11b^+^F4/80^+^ MΦ maturated from bone marrow-induced MO-MDSC treated with lentivirus-transfected Smad3/Ctrl for 5 days under 10ng/mL M-CSF condition. (H) Cell numbers and FCM analysis of the proportions of CD11b^+^F4/80^+^ MΦ maturated from Lewis model-derived MO-MDSC treated with 2μM DMSO/SIS3 for5 days under 2μM ATRA, 10ng/mL GM-CSF and 20% TCCM condition. (I) Western blot analysis of the Smad3 expressions in Lewis model-derived MO-MDSC treated with 2μM DMSO/ATRA for 48 hours. (J) qRT-PCR analysis of the Smad3 expressions in Lewis model-derived MO-MDSC treated with 2μM DMSO/ATRA for 24 hours. (K, L) Cell numbers and FCM analysis of the proportions of CD11c^+^MHCII^+^ DC maturated from bone marrow-induced MO-MDSC treated with 2μM DMSO/SIS3 for 6 days under 20ng/mL GM-CSF and 10ng/mL IL-4 condition. (M, N) FCM analysis of the proportions of CD11c^+^MHCII^+^ DC maturated from bone marrow-induced MO-MDSC and MHCII expression treated with lentivirus-transfected Smad3/Ctrl for 6 days under 10 ng/mL GM-CSF and 2.5 ng/mL IL-4 condition. Error bars of statistic graphs represent mean ± SD, n=3. p values were determined using two sides unpaired t-test.

**Supplementary Fig. 4.**

(A) Analysis of the Arg-1 activity, NO concentration and ROS expression in 0 (Ctrl) or 10ng/mL Tgfβ1(Tgfβ) induced MDSC combined with GM-CSF and IL-6 for 4 days. (B, C) FCM analysis of the proportions of MDSC subgroups and cell numbers after 0/10/20/50 ng/mL Tgfβ1 treatment in MDSC induction from bone marrow cells by TCCM for 4 days. (D) The total cell numbers and MDSC subgroup cell numbers after MDSC induction from bone marrow cells with or without 10μM anti-IgG/Tgfβ for 4 days by TCCM. (E) FCM analysis of the proportions of MDSC and subgroups after MDSC induction from bone marrow cell treated with lentivirus transfected Smad3/Ctrl for 4 days by TCCM. (E) FCM analysis of MHCII expression during MDSC induction from bone marrow treated with lentivirus transfected Smad3/Ctrl for 4 days by TCCM. (G, H) Cell numbers and FCM analysis of the proportions of MDSC and subgroups with DMSO/SIS3 treatment during MDSC induction by TCCM for 4 days. (I) Western blot identified the inhibition of 2μM SIS3 treatment to Smad3 activation in BMC for 2 hours under TCCM condition. (J) FCM analysis of the proportions of MDSC and subgroups with or without 10μM anti-IgG/Tgfβ1 in Lv-Smad3/Lv-Ctrl groups of MDSC induction by TCCM. (K, L) FCM analysis of MΦ and DC proportions in IgG, anti-Tgfβ1 and anti-Tgfβ1 adding SIS3 treatment group respectively in MΦ and DC induction system from BMC. Error bars of statistic graphs represent mean ± SD, n=3. For A, E-H and J, p values were determined using two sides unpaired t-test; For B-D and K-L, p values were determined using one-way ANOVA with Tukey test. *, p< 0.05; **, p< 0.01; ***, p< 0.001; ****, p< 0.0001; ns, no significant.

**Supplementary Fig. 5.**

(A, B) FCM analysis of Ki67 expressions in IgG, anti-Tgfβ1 and anti-Tgfβ1 adding SIS3 treatment group respectively in MΦ and DC induction system from BMC. Error bars represent mean ± SD, n=3. p values were determined using one-way ANOVA with Tukey test. *, p< 0.05; ns, no significant.

**Supplementary Fig. 6.**

(A) qRT-PCR analysis of Smad3 expressions in CD11b^+^Ly6C^+^ cells derived from spleens of wide-type mice, CD11b^+^Ly6C^+^ MO-MDSC derived from spleens of CT26-bearing mice and peritoneal macrophages (pMΦ) derived from wide-type mice (B) m^6^A modifications predicted by RMBase 2.0 database (<https://rna.sysu.edu.cn/rmbase/>) on Smad3 transcripts. (C) Detection of m^6^A levels on Smad3 mRNA in control and TCCM-treated groups for 48 hours through MeRIP-qPCR. (D) Western blot analysis of the Mettl3 and Smad3 expressions in tumor MO-MDSC treated with 2μM ATRA for 24 hours. (E) Western blot analysis of the Mettl3 and Smad3 expressions in tumor MO-MDSC and the ATRA-induced MΦ. (F) RIP-qPCR analysis of the binding of Ythdf1/3 protein with Smad3 mRNA in tumor MO-MDSC. Error bars represent mean ± SD from 3 independent experiments (A, C, F). For A and C, p values were determined using one-way ANOVA with Tukey test; For F, p values were determined using two sides unpaired t-test. *, p< 0.05; ***, p< 0.001; ****, p< 0.0001; ns, no significant.

**Supplementary Fig. 7.**

(A) TCGA database analysis of the expressions of Smad3 in colorectal adenocarcinoma (COAD) patients ([UALCAN](https://ualcan.path.uab.edu/cgi-bin/ualcan-res.pl)). (B) TCGA database analysis of the expressions of Mettl3 in colorectal adenocarcinoma (COAD) patients ([UALCAN](https://ualcan.path.uab.edu/cgi-bin/ualcan-res.pl)). (C) Analysis of the correlation of Smad3 and HLA-DR expressions in PBMC-derived CD11b^+^ myeloid cells of colon cancer patients. (D) GEPIA database analysis of the expressions of Smad3 in monocytes and neutrophils within tumor tissues of colon cancer patients, normal colon tissues and bone marrow of healthy individuals ([GEPIA2021](http://gepia2021.cancer-pku.cn/)).
