# Supplementary material for: Downregulated Smad3 signaling impairs the maturation of MO-MDSC in colorectal cancer
Source: Cell Death Dis. 2025 Dec 8;16(1):880. doi: 10.1038/s41419-025-08228-1 (PMC12686534; doi:10.1038/s41419-025-08228-1)
Supplement: Supplementary file 3 — Supplementary tables [file 41419_2025_8228_MOESM3_ESM.docx]

**Table S1. Differential expression of Smad3 in HLADR^-^CD33^+^CD11b^+^ cells in PBMC from CRC patients and tumor-free individuals.**

|  | | **Tumor-free** | **CRC** | **Smad3 expression (Mean±SEM)** | | **P value** |
| --- | --- | --- | --- | --- | --- | --- |
|  |  |  |  | **Tumor-free** | **CRC** |  |
| **Total (n)** | | **29** | **29** | **77019±3000** | **27229±3071** | ***P<0.0001*** |
| **Age (years)** | **Mean±SEM** | **65.34±1.62** | **62.93±1.70** |  |  | ***0.346*** |
|  | **≤ 60** | **19** | **13** | **77539±4065** | **26600±5643** | ***P<0.0001*** |
|  | **≥ 60** | **10** | **16** | **76031±4274** | **27740±3343** | ***P<0.0001*** |
|  | **P value** |  |  | **0.816** | **0.858** |  |
| **Sex** | **Female** | **12** | **8** | **77431±4607** | **27136±7995** | ***P<0.0001*** |
|  | **Male** | **17** | **21** | **76728±4070** | **27264±3116** | ***P<0.0001*** |
|  | **P value** |  |  | **0.911** | **0.985** |  |
| **TNM stage** | **I** |  | **3** |  | **58212±9639** |  |
|  | **II** |  | **10** |  | **35910±762.6** |  |
|  | **III** |  | **8** |  | **24663±1313** |  |
|  | **IV** |  | **8** |  | **7324±1314** |  |
| **Treatment** | **Surgery** **(yes/no)** |  | **14/15** |  | **22326±4397**  **/31805±4076** | ***0.125*** |
|  | **Chemotherapy (yes/no)** |  | **4/25** |  | **17549±6555**  **/27113±3302** | ***0.296*** |
|  | **Others** |  |  |  |  |  |

**Table S2. Clinical characteristics of CRC patients.**

| **Characteristics** |  | **CRC patients** |
| --- | --- | --- |
| **Total (n)** |  | **28** |
| **Age(years)** | **Mean±SEM** | **62.93±9.17** |
|  | **≤ 60** | **14** |
|  | **≥ 60** | **18** |
| **Sex** | **Female** | **9** |
|  | **Male** | **23** |
| **TNM stage** | **I** | **3** |
|  | **II** | **10** |
|  | **III** | **11** |
|  | **IV** | **8** |
| **Treatment** | **Surgery (yes/no)** | **14/18** |
|  | **Chemotherapy (yes/no)** | **4/28** |
|  | **Others** |  |
| **Location** | **Colon** | **20** |
|  | **Rectal** | **12** |

**Table S3. Clinical characteristics of CRC patients.**

| **Characteristics** |  | **CRC patients** |
| --- | --- | --- |
| **Total (n)** |  | **28** |
| **Age(years)** | **Mean±SEM** | **56.5±1.99** |
|  | **≤ 60** | **18** |
|  | **≥ 60** | **10** |
| **Sex** | **Female** | **13** |
|  | **Male** | **15** |
| **TNM stage** | **I** | **7** |
|  | **II** | **11** |
|  | **III** | **6** |
|  | **IV** | **4** |
| **Treatment** |  | **no treated** |
| **Location** | **Colon** | **21** |
|  | **Rectal** | **7** |

**Table S4. Clinical characteristics of patients with CRC, breast cancer and ovarian cancer.**

| **Characteristics** |  | **CRC** **patients** | **Breast cancer patients** | **Ovarian cancer patients** |
| --- | --- | --- | --- | --- |
| **Total (n)** |  | **22** | **20** | **17** |
| **Age(years)** | **Mean±SEM** | **67.27±1.83** | **55.75±2.88** | **61.88±2.47** |
|  | **≤ 60** | **7** |  | **6** |
|  | **≥ 60** | **15** |  | **11** |
| **Sex** | **Female** | **7** | **20** | **17** |
|  | **Male** | **15** |  |  |
| **TNM stage** | **I** | **2** | **7** | **0** |
|  | **II** | **6** | **11** | **3** |
|  | **III** | **9** | **2** | **10** |
|  | **IV** | **5** | **0** | **4** |
| **Treatment** |  | **no treated** | **no treated** | **no treated** |
| **Location** | **Colon** | **17** |  |  |
|  | **Rectal** | **5** |  |  |

**Table S5 siRNA sequences.**

| **siRNAs** | **Sequences** |
| --- | --- |
| **siMettl3** | Forward-Primer：5’-CCUCCAAGAUGAUGCACAUTT -3’  Reverse-Primer：5’- AUGUGCAUCAUCUUGGAGGTT-3’ |

**Table S****6 List of antibodies usage.**

| Antibody Name | Producer | Cas.No | Dilution |
| --- | --- | --- | --- |
| Anti-p-PI3K | CST | 17366 | 1:1000 |
| Anti-PI3K | CST | 4292 | 1:1000 |
| Anti-p-AKT (Ser473) | Proteintech | 66444-1-Ig | 1:1000 |
| Anti-AKT | Proteintech | 10176-2-AP | 1:1000 |
| Anti-Smad3 | abcam | ab40854 | 1:3000 |
| Anti-p-Smad3 (S423+S425) | abcam | ab52903 | 1:3000 |
| Anti-Smad2 | CST | 5339 | 1:1000 |
| Anti-p-Smad2 (S465+S467) | CST | 18338 | 1:1000 |
| Anti-Mettl3 | abcam | ab195352 | 1:3000 |
| Anti-Ythdf2 | abcam | Ab246514 | 1:3000 |
| Anti-Ythdf1 | Preteintech | 17479-1-AP | 1:1000 |
| Anti-Ythdf3 | Preteintech | 25537-1-AP | 1:1000 |
| Anti-p65 | CST | 8242S | 1:1000 |
| Anti-p-p65 | CST | 3033S | 1:1000 |
| β-Actin Mouse mAb | Abclonol | AC004 | 1:10000 |
| β-Actin Rabbit mAb | Abclonol | AC026 | 1:10000 |
| Anti-Rat IgG (H+L) | Abclonol | AS028 | 1:500 |
| Goat Anti-Rabbit IgG(H+L) HRP | Multi science | GAR007 | 1:10000 |
| Goat Anti-Mouse IgG(H+L) HRP | Multi science | GAM007 | 1:10000 |

**Table S7 Primer sequences used in qRT-PCR assays.**

| Gene Name | Forward Primer (5’-3’) | Reverse Primer (5’-3’) |
| --- | --- | --- |
| Smad3 | ATTCCATTCCCGAGAACACTAA | TAGGTCCAAGTTATTGTGTGCT |
| Arg-1 | GCTGGTCTGCTGGAAAAACTT | AGGGGAGTGTTGATGTCAGTGT |
| NOS2 | GAGCCCTCAGCAGCATCCAT | GGTGAGGGCTTGGCTGAGTG |
| Hrpt1 | CCGAGGATTTGGAAAAAGTGTT | CATCTCCTTCATGACATCTCGA |
| Tgfβ1 | CCAGATCCTGTCCAAACTAAGG | CTCTTTAGCATAGTAGTCCGCT |
| Tgfβ2 | CTCGACATGGATCAGTTTATGC | ATAAACCTCCTTGGCGTAGTAC |
| TgfβRI | CATTGCTGGTCCAGTCTGCTTCG | TGGTGAATGACAGTGCGGTTATGG |
| TgfβRII | GACCTCAAGAGCTCTAACATCC | GTCATCCACAGACAGAGTAGG |
| Mettl3 | CGCTGCCTCCGATGTTGATCTG | CTGACTGACCTTCTTGCTCTGCTG |
