# Supplementary material for: Downregulated Smad3 signaling impairs the maturation of MO-MDSC in colorectal cancer
Source: Cell Death Dis. 2025 Dec 8;16(1):880. doi: 10.1038/s41419-025-08228-1 (PMC12686534; doi:10.1038/s41419-025-08228-1)
Supplement: Supplementary file 4 — Supplemental Material 1-cell line authentication [file 41419_2025_8228_MOESM4_ESM.pdf]

Service Report: CT26.WT Cells

C311 Mouse Cell Line Authentication

Applied Biological Materials Inc.
B14 National University Science Park
Jingkou District
Zhenjiang, Jiangsu
212009 China
info@abmGood.com
www.abmGood.com

Service Description :

Order Number: T9061
Cell Name: CT26.WT
Species: Mouse
Samples Number: ⩾1x 10^6 cells

Test requirements:

The cell samples were tested and identified for any potential cross-contamination by comparing DNA STR data with ATCC, DSMZ, JCRB and RIKEN databases.

Services/Tests Performed:

DNA was extracted using the genomic extraction kit from Axygen and amplified by 10-STR amplification. The STR loci and sex gene Amelogenin were detected on the ABI 3730XL genetic analyzer.

Test results:

Sample Number	Multiple allele Analysis	Matched Cell Line	Cell Bank	EV Value	Matching Result
CT26.WT	NO	CT26.WT	DSMZ	1.0	**exactlly matched**

Sample genotype test results

- Multiple alleles refer to three or more alternative forms of a gene (alleles) that occupy the same locus.
- The results of genotyping were good.

This product is for research use only and is not intended for therapeutic or diagnostic applications. Please contact a technical service representative for more information.

Annex I.

Genotyping results of STR loci and Amelogenin loci of cells							
Loci	Send cell STR information				Cell bank cell STR information		
	Test cell name：CT26.WT				Cell names in stock：CT26.WT		
	Allele1	Allele2	Allele3	Allele4	Allele1	Allele2	Allele3
4-2	242.87 【21.3】				21.3		
5-5	336.24 【14】				14		
6-4	300.48 【18】	308.44 【20】			18	20	
6-7	336.17 【12】				12		
9-2	221.95 【15】				15		
12-1	226.28 【16】	230.41 【17】			16	17	
15-3	197.06 【21.3】	201.18 【22.3】			21.3	22.3	
18-3	164.64 【19】	168.78 【20】			19	20	
X-1	400.82 【25】	404.87 【26】			25	26	
D4S2408							

This product is for research use only and is not intended for therapeutic or diagnostic applications. Please contact a technical service representative for more information.

Figure II

Genotyping of STR and Amelogenin loci in cells

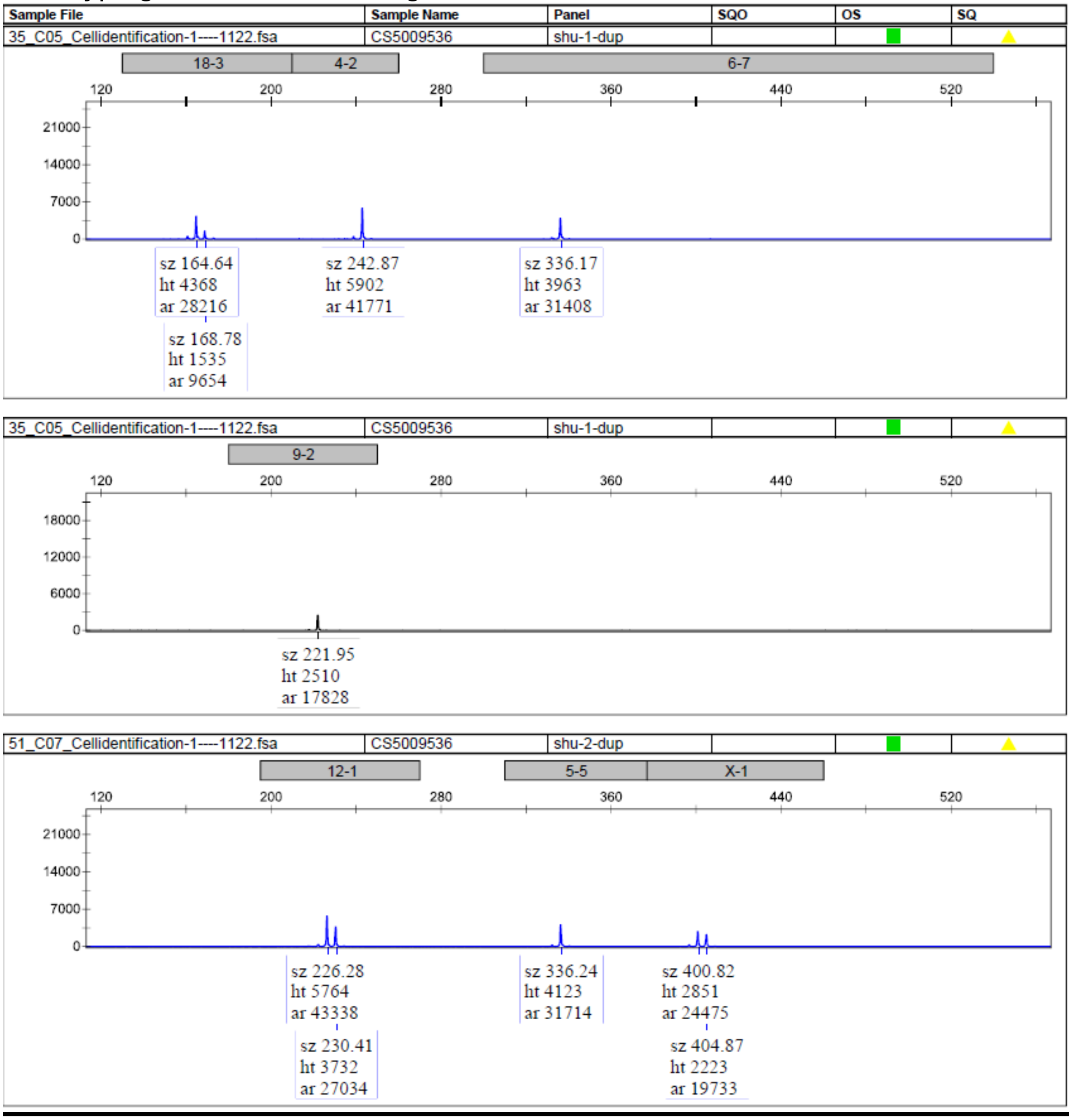

Conclusion:

CT26.WT: The genotype of this cell line has a **exactlly matched** to an existing cell bank database. The EXPASY database shows that the cell name is **CT26.WT**, with the corresponding cell number **CVCL_7256** . **No multiple alleles were found** in this cell line.

Note: We use DSMZ tools for cell line comparison, which includes STR information of 2455 cell lines archived from ATCC, DSMZ, JCRB and RIKEN databases. If the cell tested is not available from the aforementioned cell banks or if it is a new cell line established by the customer, the comparison will not be possible. The customer shall compare with other databases on their own according to the genotyping results.

Disclaimer:

The results contained in this report were generated in a research lab that is not an accredited or licensed clinical laboratory. The results are provided for research purposes only. The underlying experiments were not performed (and these results are not provided) for the purposes of obtaining information for diagnosis, prophylaxis, or treatment.

Acknowledgement:

Thank you for choosing abm as your authentication service provider. It is our goal to provide you with the best customer experience in the world. Please do not hesitate to contact us should you need further assistance analyzing your data or other services. We are grateful to be a part of your scientific exploration and we look forward to serving you again.

Certificate of Analysis

Submitted by:
Quality Control Personnel ID No.: 0246
Quality Assurance Personal ID No.: 0079

Applied Biological Materials Inc.
B14 National University Science Park, Jingkou District ,Zhenjiang, Jiangsu,212009 China

Product Description

**Product Name:**	CT26.WT Cells
**Cat. Number:**	T9061
**Lot Number:**	B24J01ES
**Species:**	Mouse (M. musculus)
**Source Organ:**	Colon
**Quantity:**	2 vials (Cryopreserved)
**Freezing Medium:**	TM024 (Cryopreservation Medium)
**Viability:**	>80%
**Cell Count:**	≥1 x 10^6 cells

Using **abm's standardized culture system and procedures; the stated value may vary depending on end user's culture conditions.*

Microbial Test Results:

	Detection Method	Range	Results
Fungal	Direct Culture	Positive / Negative	Negative
Bacterial	Direct Culture	Positive / Negative	Negative
Mycoplasma	PCR	Positive / Negative	Negative

Approval:

Approved by: Micheal

Approved Personnel ID No.: 0246

Approved Date: 4/18/2024

This product is for research use only and is not intended for therapeutic or diagnostic applications.
Please contact a technical service representative for more information.
