# Supplementary material for: Downregulated Smad3 signaling impairs the maturation of MO-MDSC in colorectal cancer
Source: Cell Death Dis. 2025 Dec 8;16(1):880. doi: 10.1038/s41419-025-08228-1 (PMC12686534; doi:10.1038/s41419-025-08228-1)

Figure 1A Western blot analysis of the Smad2/3 expressions in wide-type-derived CD11b+Gr-1+ cells (WT) and CT26-derived spleen CD11b+Gr-1+ MDSC (Tu).

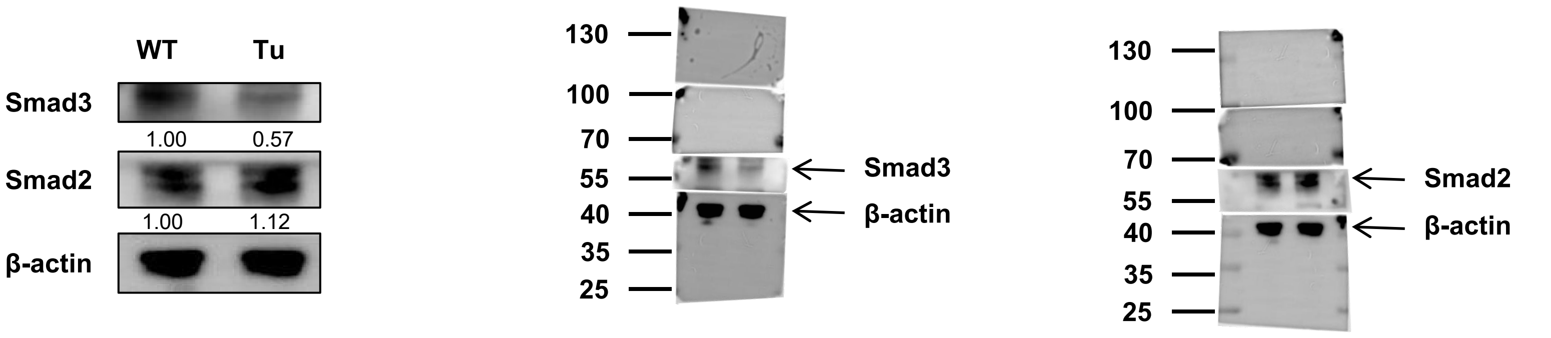

Figure 1D Western blot analysis of the Smad3 expressions in wide-type-derived CD11b+Gr-1+ cells (WT) and MDSC with tumor progression of CT26 mouse models respectively at early (8d), middle (18d) and late stage (28d).

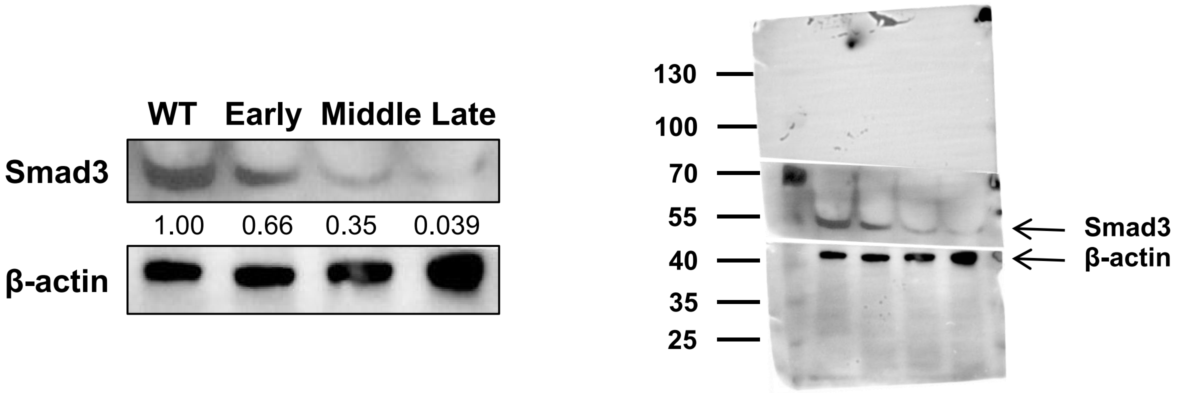

Figure 1L Western blot analysis of the Smad3 expressions in initial bone marrow cells (BMC) and BMC-induced MΦ , DC and MO-MDSC.

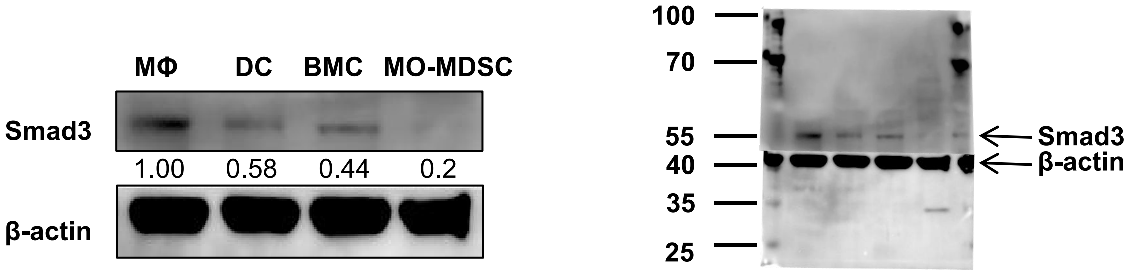

Figure S1E Western blot analysis of the expressions of Smad2/3 in BMC and the TCCM-induced BM-MDSC.

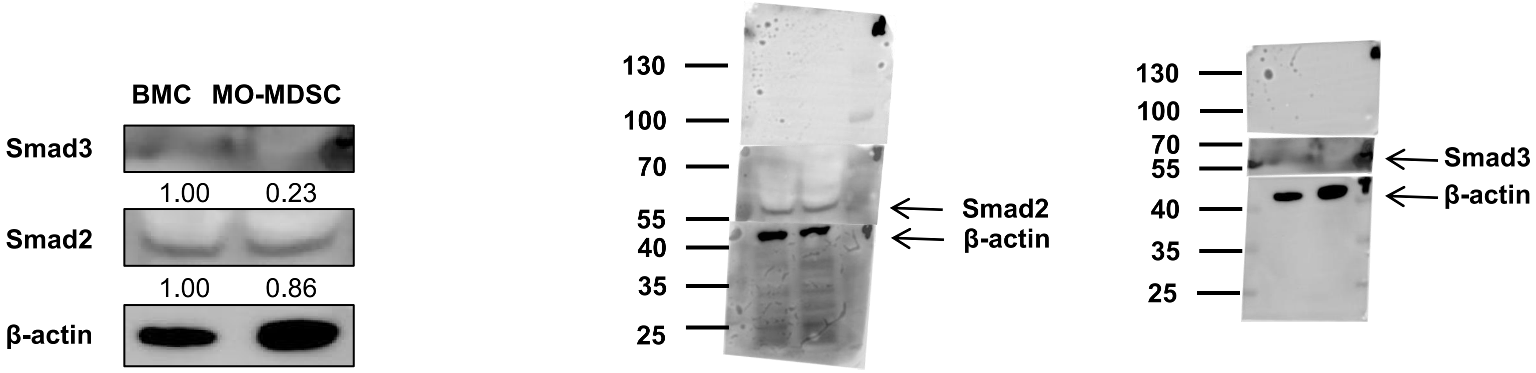

FigureS1M Western blot analysis of the expressions of Smad3 during DC induction from BMC.

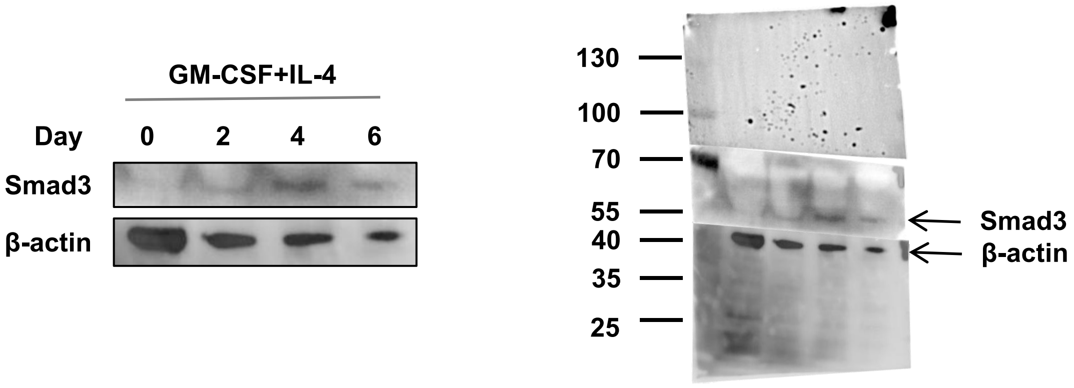

FigureS1N Western blot analysis of the expressions of Smad3 during MΦ induction from BMC.

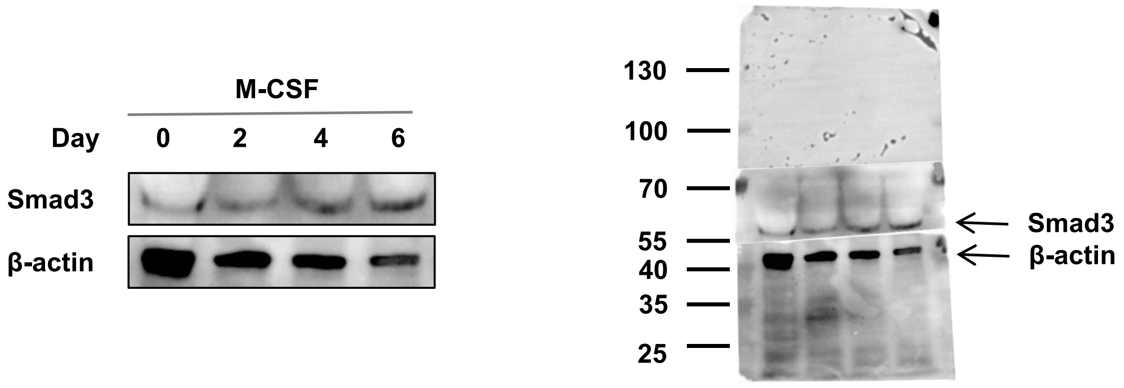

Figure S1O Western blot analysis of the Smad3 expressions in wide-type mice-derived CD11b⁺Ly6C⁺ cells (WT-CD11b⁺Ly6C⁺), CT26-isolated spleen CD11b⁺Ly6C⁺ MDSC (Tu-MO-MDSC) and wide-type mice-derived peritoneal macrophages (pMΦ).

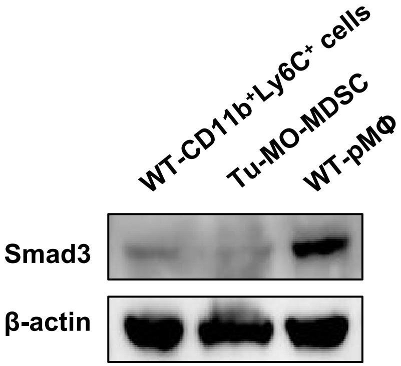

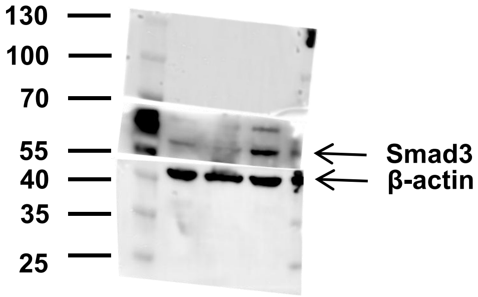

Figure 3I Western blot analysis of the Smad3 expressions in tumor MO-MDSC treated with 2μM DMSO/ATRA for 48 hours.

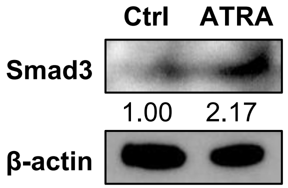

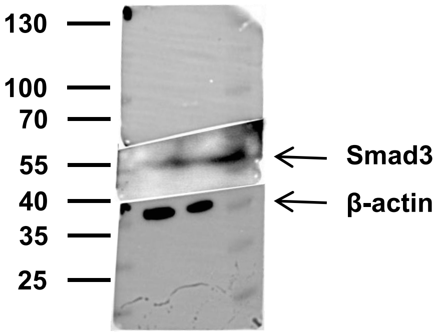

Figure S3C Western blot analysis of the Smad3 expressions in tumor MO-MDSC and the M-CSF-induced MΦ (iMΦ).

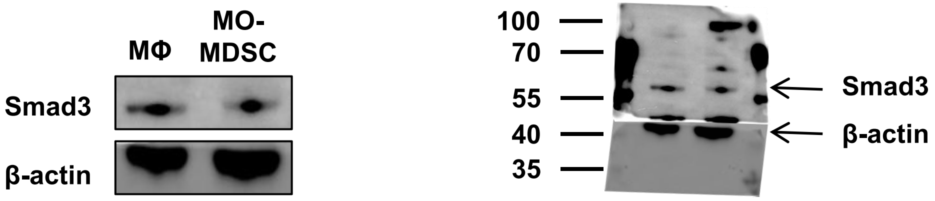

Figure S3I Western blot analysis of the Smad3 expressions in Lewis model-derived MO-MDSC treated with 2μM DMSO/ATRA for 48 hours.

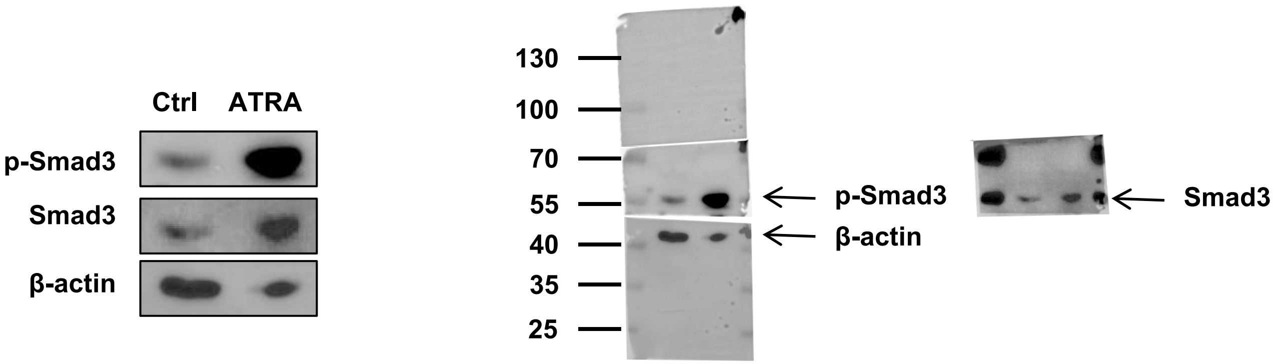

Figure 4E Western blot analysis of the Smad3 expression in BMC with or without TCCM treatment for 48 hours.

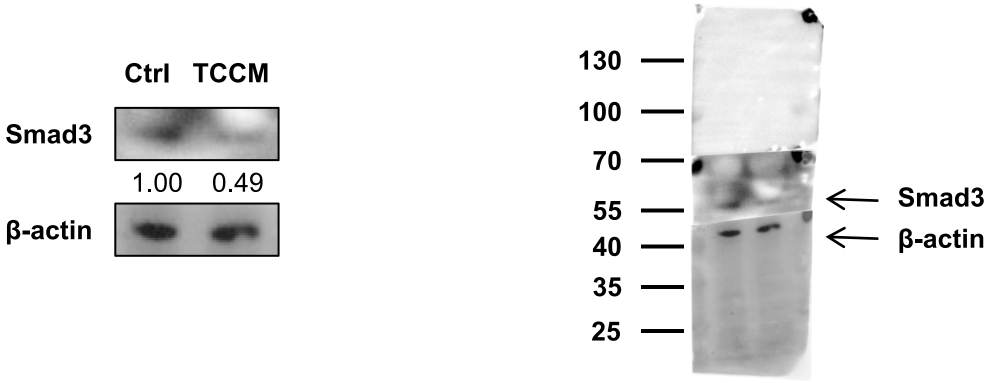

Figure 4I Western blot analysis of the expressions of Smad3 during MO-MDSC induction from BMC at 0,1,2,4 days.

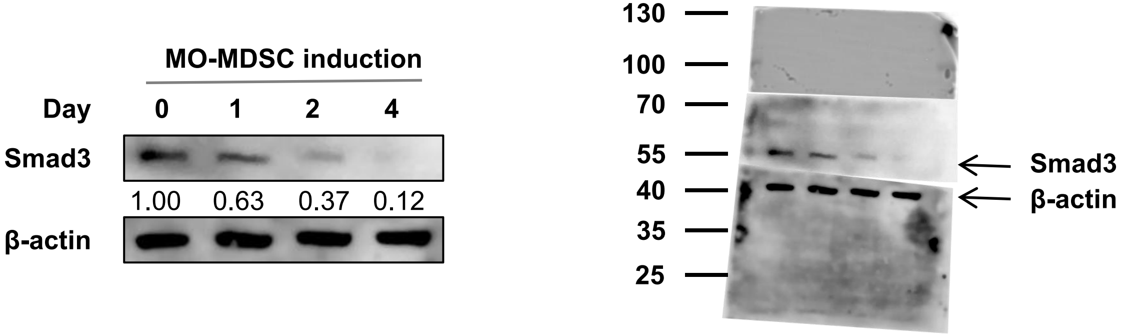

Figure S4I Western blot identified the inhibition of 2μM SIS3 treatment to Smad3 activation in BMC for 2 hours under TCCM condition.

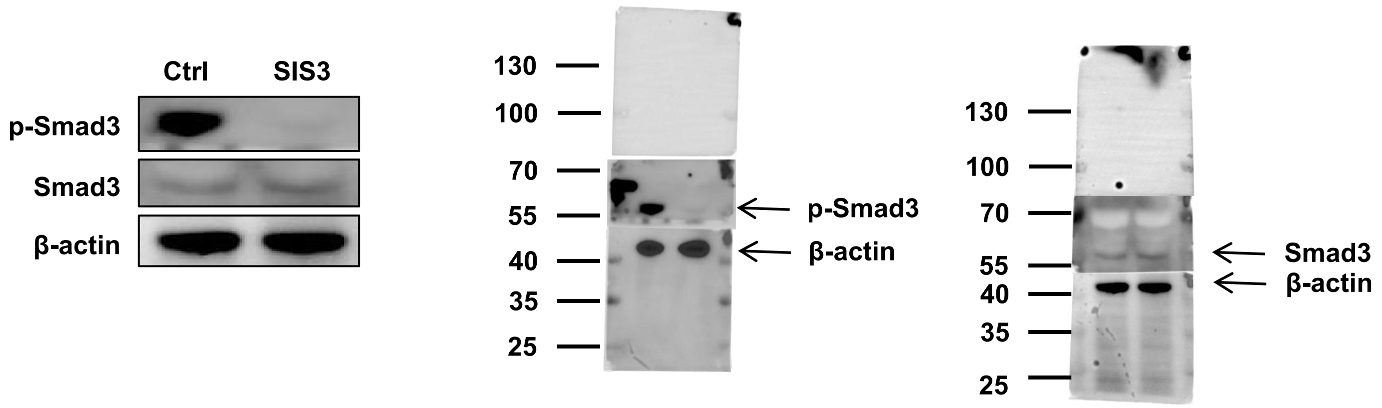

Figure 5A Western blot analysis of the activations of PI3K/AKT pathway in BMC and BM-induced MO-MDSC by TCCM with or without 20ng/mL Tgfβ1 treatment for 2 hours.

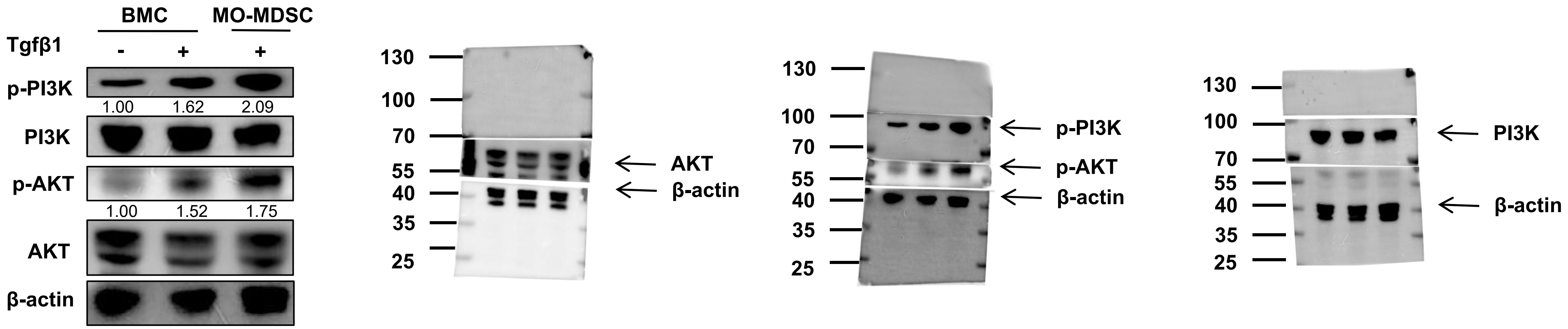

Figure 5B Western blot analysis of the activations of PI3K/AKT pathway in BM-induced MO-MDSC with 0.2μM DMSO/Wortm treatment for 2 hours under Tgfβ1 condition.

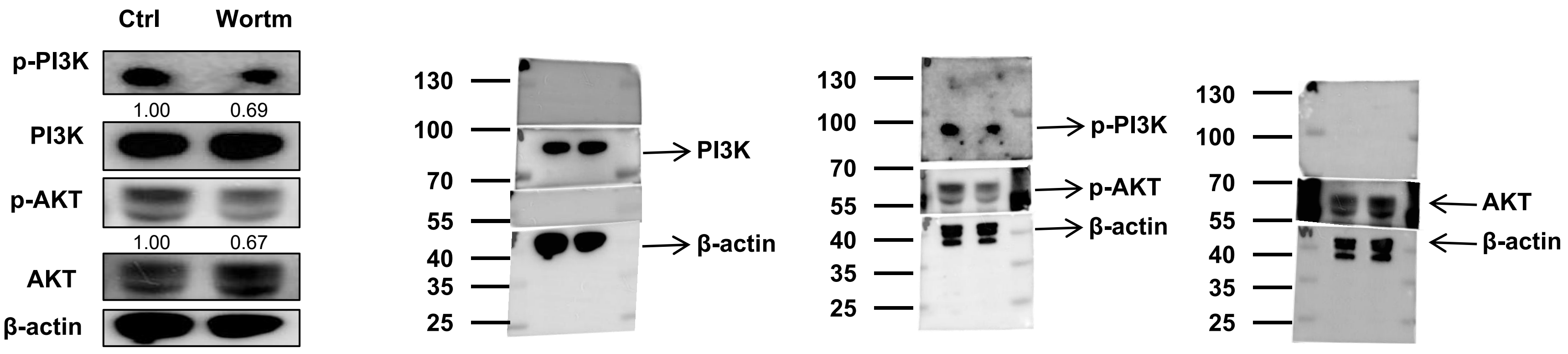

Figure 5H Western blot analysis of the PI3K activation in BM-induced MO-MDSC after Lv-Ctrl/Smad3 transfection for 48 hours under Tgfβ1 condition.

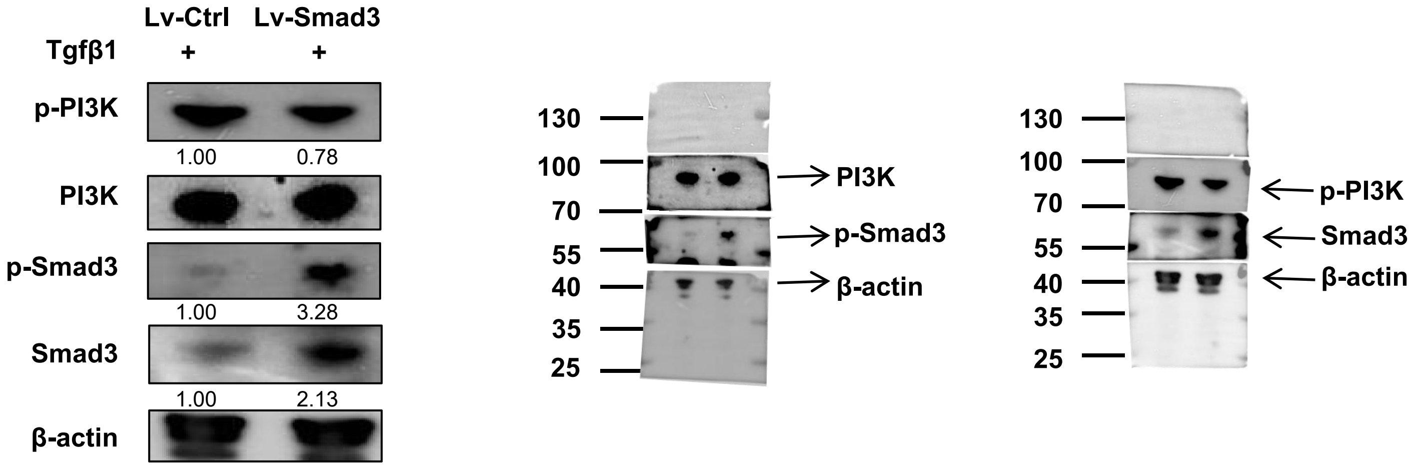

Figure 6C Western blot analysis of the Smad3 expressions in tumor MO-MDSC with or without 5μM STM2457 treatment for 6/12/24/48/72 hours.

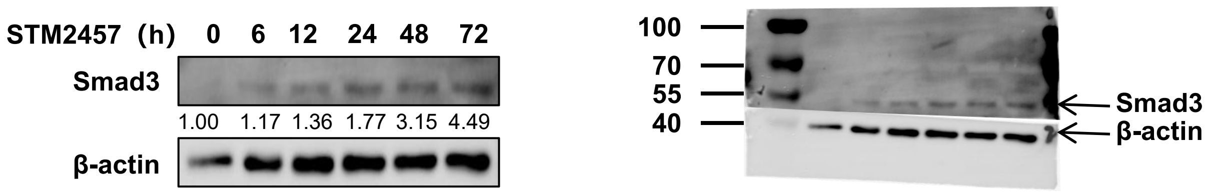

Figure 6G Western blot analysis of the Mettl3 expression in initial bone marrow cells (BMC) and bone marrow induced MO-MDSC by TCCM (BM-MO-MDSC).

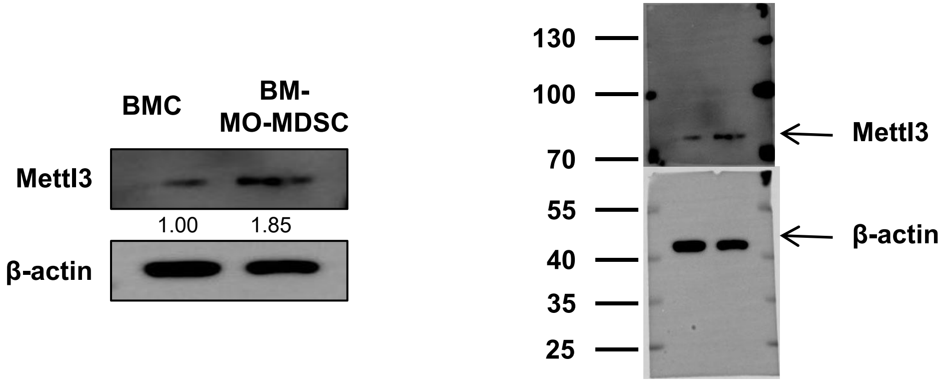

Figure 6H Western blot analysis of the Mettl3 expressions in wide-type mice-derived pMΦ, tumor-derived MO-MDSC and wide-type mice-derived $CD11b^{+}Ly6C^{+}$ cells.

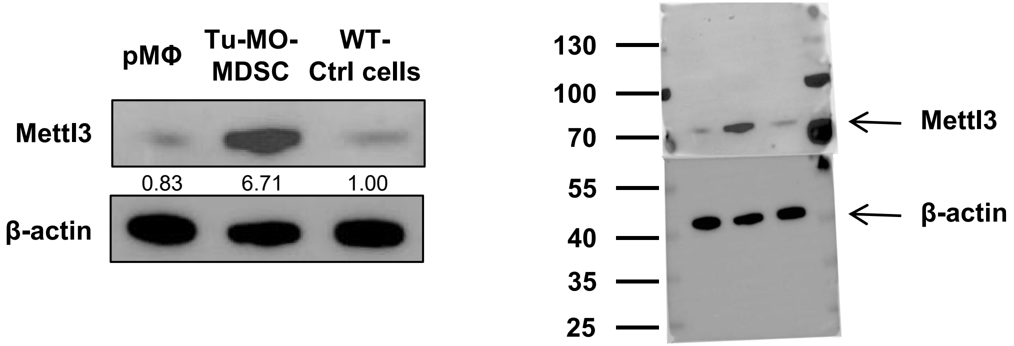

Figure S6D Western blot analysis of the Mettl3 and Smad3 expressions in tumor MO-MDSC treated with 2μM ATRA for 24 hours.

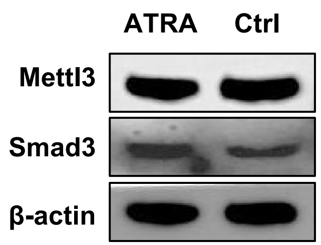

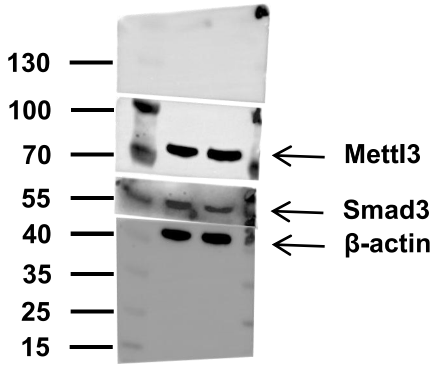

Figure S6E Western blot analysis of the Mettl3 and Smad3 expressions in tumor MO-MDSC and the ATRA-induced MΦ.

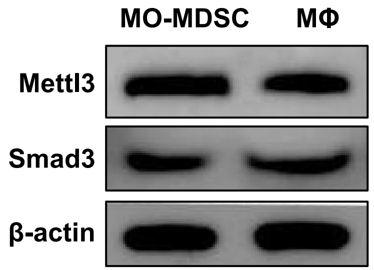

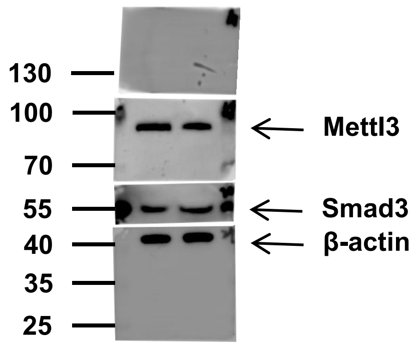

Supplement: Supplementary file 5 — Supplemental Material 2-Primary Western blot picture [file 41419_2025_8228_MOESM5_ESM.pdf]
